# 3D Printing of Bicontinuous Nanoparticle‐Stabilized Emulsion Gels via Co‐Solvent Removal

**DOI:** 10.1002/smll.202504718

**Published:** 2025-11-03

**Authors:** Philip R. Iaccarino, Damilola Lawal, Jordan R. Raney, Kathleen J. Stebe, Daeyeon Lee

**Affiliations:** ^1^ Department of Chemical and Biomolecular Engineering University of Pennsylvania 220 South 33rd St. Philadelphia PA 19104‐6315 USA; ^2^ Department of Materials Science and Engineering University of Pennsylvania 3231 Walnut St. Philadelphia PA 19104‐6272 USA; ^3^ Department of Mechanical Engineering and Applied Mechanics University of Pennsylvania 220 South 33rd St. Philadelphia PA 19104‐6315 USA

**Keywords:** bigels, bijels, direct ink write, hierarchical materials, multiphasic inks

## Abstract

Bicontinuous emulsion gels are mixtures with interpenetrating arrangements of two immiscible liquids stabilized with particles. The structures of such gels are readily made into simple macroscale geometries, like sheets and fibers; however, achieving more complex macroscopic structures while maintaining control over microscopic features and morphological bicontinuity remains a challenge. In this study, the ability to fabricate complex 3D structures of bicontinuous emulsion gels using direct ink writing (DIW) is demonstrated. The emulsion precursors are formulated with a mixture of hydrophilic and hydrophobic fumed silica particles; these precursors exhibit shear‐thinning and yield stress behavior necessary for DIW. The thixotropic nature of the precursor further promotes the formation of bicontinuous emulsion gels through vaporization‐induced phase separation and stabilization through both interfacial jamming and bulk stabilization mechanisms. This fabrication technique enables the creation of functional bicontinuous structures with complex architectures, paving the way for application in biomedical implants, catalytic reactors, and beyond.

## Graphical abstract

A DIW approach is developed to 3D print bicontinuous nanoparticle‐stabilized emulsion gels. By combining hydrophilic and hydrophobic fumed silica, the precursor acquires the yield stress and thixotropy required for printing. Vaporization‐induced phase separation produces submicrometer bicontinuous domains, enabling the formation of hierarchical porous materials with customizable geometries for advanced separation and biomedical applications.

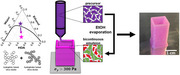

## 1 Introduction

Mixtures of dissimilar liquids, like oil and water, are inherently unstable, leading to complete phase separation in their equilibrium state. The addition of stabilizers into these mixtures enables the formation of a kinetically trapped emulsion in a non‐equilibrium state. When particles are used as stabilizers, two distinct classes of emulsions can be created: Pickering emulsions or bicontinuous emulsions.^[^ ^1^, ^2^ ^]^ In the former, one phase forms discrete droplets dispersed in the other phase. In the latter, both phases are continuous and form an interpenetrating percolating liquid network throughout the emulsion. Bicontinuous emulsion gels possess unique properties, making them potentially useful in various applications. The bicontinuous configuration allows for independent transport of oil‐ or water‐soluble species through their respective channels, while the high surface area of the interwoven phases promotes transport between the two phases.^[^ ^3^ ^]^ Bicontinuous emulsion gels, with one aqueous and one organic domain, are classified based on their stabilization mechanism. Examples of these materials include bicontinuous interfacially jammed emulsion gels (bijels), stabilized by interfacial particle attachment and subsequent jamming,^[^ ^4^, ^5^, ^6^, ^7^ ^]^ and biphasic gels (bigels), stabilized through bulk nanoparticle jamming within the two phases.^[^ ^8^, ^9^, ^10^, ^11^ ^]^ Attractive or repulsive particle networks have also shown to stabilize bicontinuous emulsion gels in bicontinuous intraphase jammed emulsion gel (bipjel) and solvent segregation driven gel (SeedGel) systems.^[^ ^12^, ^13^ ^]^

Various methods have been developed for the fabrication of bicontinuous emulsion gels. For example, bicontinuous emulsions are often produced using binary liquid mixtures with a lower critical solution temperature, such as a water‐lutidine mixture. A temperature quench triggers phase separation through the critical point of the phase diagram, initiating spinodal phase separation and generating a bicontinuous morphology. Bijels, bipjels, and SeedGels have been fabricated using this technique; the stabilization mechanisms across emulsion types depend on the particle chemistry.^[^ ^4^, ^12^, ^13^ ^]^ For bijels, complementary approaches exploit a single phase, ternary mixture of co‐solvent, organic and aqueous components. Removal of co‐solvent near the system's critical point initiates spinodal decomposition, leading to the formation of bijels that are stabilized through the interfacial attachment and jamming of neutrally wetting particles. Co‐solvent removal can be achieved either by mass transfer into an external aqueous phase, triggering solvent‐transfer induced phase separation (STRIPS),^[^ ^5^ ^]^ or by vaporization into the surrounding gas phase, leading to vaporization‐induced phase separation (VIPS).^[^ ^6^ ^]^ Bicontinuous structures in the form of bigels and bijels can be fabricated by directly mixing two immiscible liquids.^[^ ^7^, ^9^ ^]^

Applications of bicontinuous emulsions exploit enhanced transport properties made possible by their structure. For example, bijels with their interfacially jammed particle layer have shown promise in applications in reactive separations and liquid‐liquid extraction.^[^ ^14^, ^15^, ^16^ ^]^ Bigels have been explored for applications in food science and cosmetics.^[^ ^10^, ^17^ ^]^ Moreover, to capitalize on the bicontinuous structure of these systems, highly porous materials with interconnected polymeric and pore phases have been formed by crosslinking one of the two liquid phases.^[^ ^18^ ^]^ Such bicontinuous materials have been developed for applications such as ultrafiltration,^[^ ^19^ ^]^ passive daytime radiative cooling coatings,^[^ ^20^ ^]^ energy storage devices,^[^ ^21^, ^22^ ^]^ and hydrogels for cell delivery systems.^[^ ^23^ ^]^

The functionality of bicontinuous emulsions depends heavily on their macroscopic geometry as well as their internal microstructures. Current fabrication techniques restrict the production of bicontinuous emulsion gels to relatively simple morphologies. Thermally quenched structures are typically limited by the shape of their container and temperature gradients. STRIPS and VIPS methods facilitate continuous manufacturing of bicontinuous emulsion gels and have been used to create bijels with a range of morphologies, such as particles,^[^ ^5^ ^]^ fibers,^[^ ^5^, ^14^, ^15^, ^16^, ^19^ ^]^ thin films,^[^ ^5^, ^6^, ^20^, ^24^ ^]^ ropes,^[^ ^25^ ^]^ and other complex 2D planar structures.^[^ ^26^ ^]^

3D printing is a promising approach to create bicontinuous emulsion gels with more complex designs, allowing for advanced integration of bicontinuous structures into engineering systems. DIW is a widely used 3D printing method in which the material, or “ink”, is extruded layer‐by‐layer onto a substrate until the desired structure is formed.^[^ ^27^ ^]^ DIW is particularly well‐suited for bicontinuous gel fabrication because it is compatible with a wide range of inks, including colloidal gels and emulsions.^[^ ^28^, ^29^ ^]^ This versatility makes DIW an excellent method for creating bicontinuous emulsion gels with complex 3D geometries, opening new possibilities for their applications.

Previous efforts to create bicontinuous hierarchical materials via extrusion‐based 3D printing have achieved varying degrees of success. Typical bicontinuous gel inks are formulated by direct mixing of aqueous and organic components, enabling the development of 3D structures such as cubic lattices with functional properties such as high electrical conductivity.^[^ ^17^, ^30^, ^31^, ^32^, ^33^, ^34^, ^35^ ^]^ In this study, we opt for a phase separation‐driven process to generate the bicontinuous emulsion structure that provides distinct advantages. For example, the generation of the bicontinuous microstructure through VIPS, particularly at the sub‐micrometer scale, does not require energy‐intensive mixing processes, making this approach more suitable for scalable manufacturing. Moreover, the morphology and the domain size can be controlled by adjusting ambient factors such as relative humidity or solvent removal rate, as well as by adjusting compositional factors,^[^ ^6^ ^]^ ensuring consistent tunability and reproducibility across operators and equipment. While STRIPS bijels have been produced through liquid‐in‐liquid printing, their macroscale geometries have been restricted to 2D planes due to the higher densities relative to the surrounding bath media.^[^ ^26^ ^]^

In this work, we present a technique to fabricate 3D, VIPS‐based bicontinuous nanoparticle‐stabilized emulsion gels via DIW. Our approach to create these hierarchical materials combines top‐down DIW fabrication to manipulate the material's centimeter‐scale macroscale geometry with bottom‐up assembly mechanisms to produce bicontinuous submicrometer domains. To do so, we design a DIW printable one‐phase precursor ink that spontaneously phase separates into a two‐phase bicontinuous emulsion gel after extrusion via ambient co‐solvent evaporation. Inspired by prior work that demonstrated the stabilization of various multiphasic structures,^[^ ^36^, ^37^, ^38^ ^]^ we introduce mixtures of hydrophilic and hydrophobic fumed silica into the emulsion precursor to modify its rheology and stabilize the bicontinuous structure. We demonstrate the effectiveness of this approach by fabricating bicontinuous emulsion gels with complex 3D morphologies and identifying the key rheological characteristics of the successful precursor ink. Additionally, we explore how variations in the precursor composition influence the resulting structure at the submicrometer scale. This printing method enables the continuous fabrication of bicontinuous nanoparticle‐stabilized emulsions with hierarchical and complex 3D geometries, allowing for the engineering of components with enhanced transport properties that promote both bulk transport within each phase and interfacial transport across phases. This ability to control the shape of bicontinuous structures is particularly valuable for applications in biomedical implants, tissue engineering, biphasic reactive separation, heat exchangers, filtration systems, and more.

## 2 Results and Discussion

**Figure 1:**
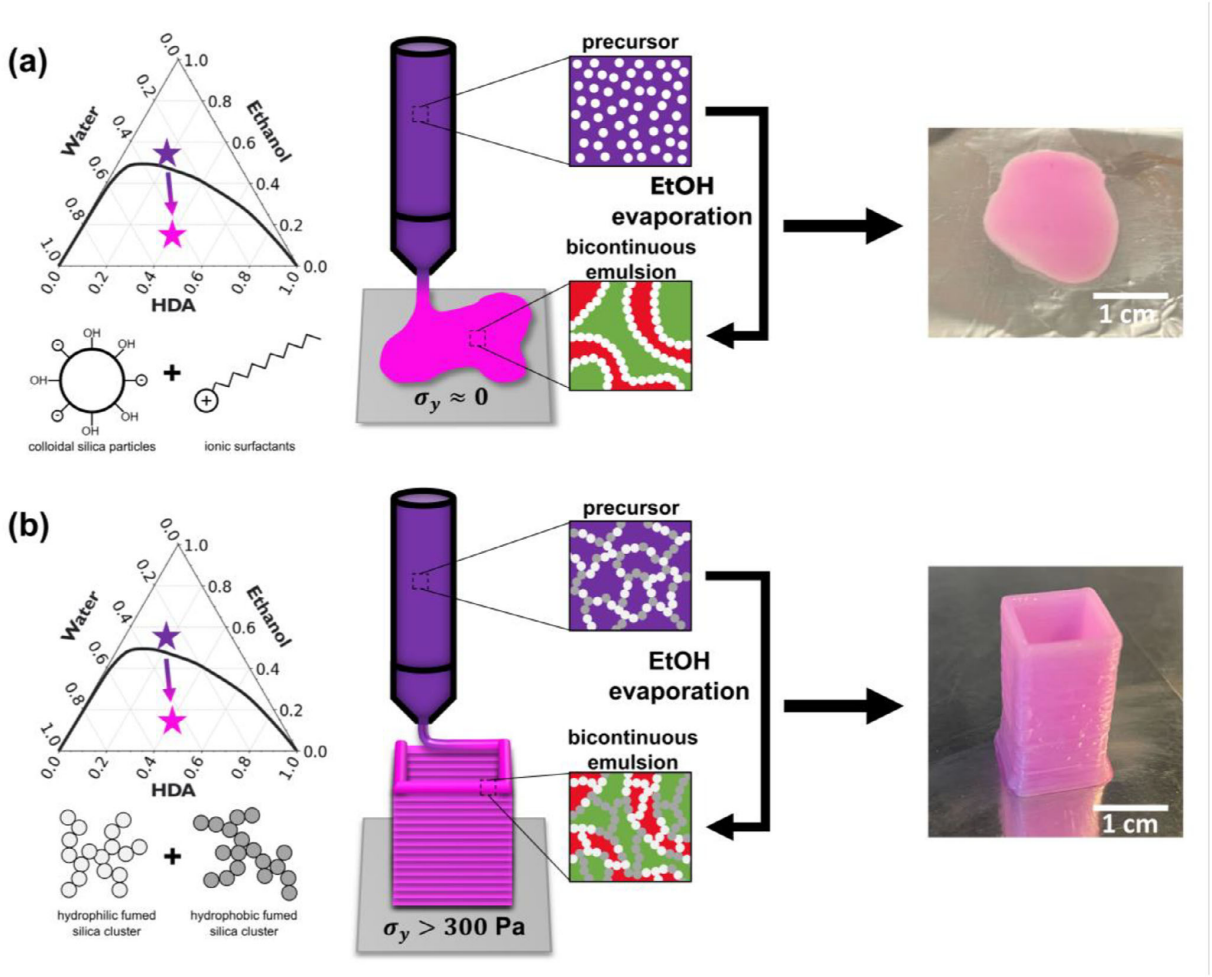
Fabrication of VIPS bicontinuous emulsion gels via DIW 3D printing. a) 3D printing fails when using conventional VIPS precursors due to their fluid‐like behavior. In these systems, surfactant‐modified silica particles are mixed into a one‐phase ternary mixture (purple), then quenched into a two‐phase region (pink) via ethanol evaporation to form particle‐stabilized bicontinuous oil‐rich phases (green) and water‐rich phases (red). b) 3D printing is successful when using fumed silica‐based emulsion precursors. In these systems, mixtures of hydrophilic (light grey) and hydrophobic (dark grey) fumed silica particles confer ink rheology crucial to successful 3D layering of material and inhibition of spreading, and also stabilize bicontinuous structures after quenching.

We use vaporization‐induced phase separation (VIPS) to produce bicontinuous emulsion gels due to this method's simplicity and versatility. Bicontinuous emulsion 3D printing via VIPS, however, requires modification of the conventional VIPS bijel precursor suspension that typically consists of an oil‐water‐solvent ternary mixture, colloidal silica particles, and surfactants. These conventional precursors spread on the substrate and are unable to support vertical layering of material due to their fluid‐like behavior. As such, the conventional VIPS bijel precursor is incompatible with 3D printing as shown in **Figure** **1**a (see Video S1, Supporting Information). We modify the conventional VIPS bijel precursor to enable DIW‐based 3D printing. Rather than using colloidal silica nanoparticles with surfactants, we use fumed silica particles to support 3D structures, as depicted in Figure 1b. These fumed silica particles provide structural stability through both interfacial jamming and bulk stabilization;^[^ ^36^, ^37^, ^38^ ^]^ this latter mechanism confers the rheological characteristics necessary to support 3D structures during printing.

The materials used to create the printable emulsion precursor include 1,6‐hexanediol diacrylate (HDA) as oil, water as an aqueous phase (pH adjusted to 3 via addition of 1 m HCl), ethanol as a co‐solvent, CAB‐O‐SIL LM‐150 hydrophilic fumed silica particles, CAB‐O‐SIL TS‐610 hydrophobic fumed silica particles, and 2‐hydroxy‐2‐methylpropiophenone (HMP) as a photoinitiator. The oil HDA is chosen because it can be polymerized under UV light in the presence of the photoinitatior HMP. Photopolymerization facilitates structural characterization via confocal laser scanning microscopy (CLSM) and scanning electron microscopy (SEM). Ethanol is an appropriate co‐solvent due to its high volatility and its ability to form a single one‐phase miscible solution with water and HDA, which are immiscible and form a two‐phase mixture at room temperature without the presence of co‐solvents. The pH of the water is adjusted to 3, as the surface chemistry of silica and its wetting behavior are dependent on the pH, and it is a well‐established condition for particle stabilization in similar systems in prior work and in many bicontinuous emulsion systems.^[^ ^37^, ^39^ ^]^ We select these two variants of fumed silica particles because they attach well to the HDA‐water interface (see Figure S1, Supporting Information); we also have previously used fumed silica in the stabilization of STRIPS bijels.^[^ ^37^ ^]^ The wetting behavior of the two fumed silica particles differs substantially; the hydrophilic variant can be dispersed in both water and HDA, whereas the hydrophobic fumed silica particles can only be suspended in HDA (see Figure S2, Supporting Information). The composition of the precursor ink is designed to satisfy two conditions: the oil‐to‐water ratio should be near the critical point of the phase diagram to promote bicontinuous phase generation upon ethanol removal, and the ratio of hydrophilic‐to‐hydrophobic fumed silica should promote formation of neutrally wetting silica clusters. Information regarding the ability of our formulation to satisfy these requirements is summarized in Supporting Information. Briefly, the ternary phase diagram is established, the critical point along the binodal line is located to promote bicontinuous emulsion formation (see Figure S3 and Table S1, Supporting Information), and a neutrally wetting particle ratio is identified to facilitate interfacial stabilization (see Figure S4, Supporting Information). Here, we also note the tendency of fumed silica to form fractal‐like clusters and aggregates, with sizes ranging from 300 to 400 nm (see Figure S5, Supporting Information).

### 2.1 Fumed Silica Particles Enable 3D Printing through Precursor Gelation

To formulate an emulsion precursor that is DIW‐compatible, we investigate the effect of hydrophilic and hydrophobic fumed silica particles on the rheological properties of the ternary liquid mixture. Ideal inks for DIW 3D printing are shear‐thinning materials with yield‐stress behavior, enabling shape retention after extrusion and the ability to support the gravitational stresses exerted by layers that are printed on top of each other. In a yield stress fluid, if stress is applied above a threshold, flow will be induced. If an ink's yield stress, storage modulus, and viscosity are not appropriately tuned, the ink is not “printable” and will spread on the substrate. Such inks cannot support vertical layering, resulting in poor shape retention.^[^ ^40^, ^41^ ^]^ For example, prior reports of non‐bicontinuous emulsion‐based DIW inks have shown printability relies on the ink's shear‐thinning behavior and its yield stress above a threshold of 200 Pa, or with a product of yield stress and storage modulus greater than 5 × 10^6^ Pa^2^.^[^ ^42^, ^43^, ^44^ ^]^

**Figure 2:**
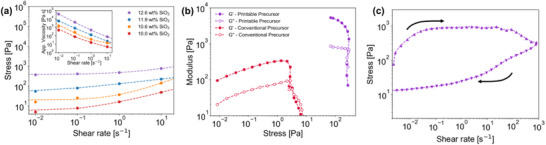
Fumed silica particles enable 3D printing through precursor gelation. a) Flow sweep rheology reveals yield stress and shear‐thinning behavior. As the fumed silica particle loading increases, both yield stress and fluid apparent viscosity increase. The dashed line represents a fit to Hershel–Bulkley model. b) Oscillatory rheology reveals gel‐like behavior of the precursor at small amplitudes and a transition to fluid‐like behavior at large amplitudes. The enhanced yield stress and storage modulus allow 3D printing, which was not possible with the conventional VIPS precursor. c) The precursor ink is thixotropic; the stress hysteresis loop shows a loss of fluid structure after exposure to high shear rates experienced during 3D printing extrusion with a partial yield stress recovery.

To determine the particle content required to impart these characteristics to the printable emulsion precursor, we perform flow‐sweep rheology of precursors with increasing particle loadings. For all silica loadings, stress increases as shear rate increases with non‐Newtonian behavior as shown in **Figure** **2**a. The flow sweep data is fit to the Hershel‐Bulkley fluid model; the model parameters are summarized in Table S2 (Supporting Information) for each silica loading. The yield stress, which can be inferred from the y‐intercept of Figure 2a, increases with particle loading. The data also indicates shear‐thinning behavior, as the Hershel‐Bulkley flow parameter is found to be below unity for each of the silica loadings. For reference, in this model, a flow parameter equal to one implies Newtonian behavior, whereas a value greater than one implies shear‐thickening behavior, and a value less than one implies shear‐thinning behavior. Apparent viscosity decreases as shear rate increases for all silica loadings, indicating shear‐thinning behavior. Further, the apparent viscosity of the precursor increases with the concentration of fumed silica in the precursor.

The precursor with a silica loading of 12.5 wt% is of particular interest for further analysis, since its flow parameters are consistent with other multiphasic (but non‐bicontinuous) complex fluids that have been used for DIW 3D printing.^[^ ^42^, ^43^, ^44^ ^]^ Prior reports have shown that the minimum yield stress to support DIW 3D printing of emulsion‐based fluids is generally ≈200 Pa in combination with shear‐thinning behavior. We find that the precursor with 12.5 wt% fumed silica has a yield stress ≈350 Pa and is shear‐thinning. We adopt this concentration of fumed silica in our precursor ink and use it to create all 3D printed bicontinuous structures presented in this manuscript, including the example shown in Figure 1b. We perform additional rheological characterization of the 12.5 wt% precursor ink to further understand its properties for 3D printing.

Small amplitude oscillatory shear (SAOS) rheology of the emulsion precursor is shown, where the precursor's storage (G′) and loss (G″) moduli are measured in response to an increasing deformation amplitude with a fixed frequency of 2π rad s^−1^ (see Figure S6a, Supporting Information). As amplitude increases, the deformation stress response of the precursor increases, influencing the moduli. In the SAOS regime, the precursor exhibits solid‐like or gel‐like behavior, indicated by the storage modulus exceeding the loss modulus by an order of magnitude, along with a linear viscoelastic region (LVR) plateau. As amplitude increases further beyond the LVR, large amplitude oscillatory shear (LAOS) reveals the precursor undergoes a gel‐to‐fluid transition, as indicated by the loss modulus overtaking the storage modulus in Figure 2b. This transition point where G″ surpasses G′ is another method to measure yield stress. This experiment reveals a precursor ink yield stress on the order of 300 Pa, which agrees with the value found previously from fitting flow sweep data to the Hershel–Bulkley model.

We also compare the behavior of the 3D‐printable fumed silica‐based emulsion gel precursor to that of the conventional colloidal silica‐based VIPS bijel precursor. The conventional bijel precursor has a negligible yield stress and a low storage modulus in the LVR, over an order of magnitude less than that of the 3D‐printable fumed silica system. The conventional bijel precursor is unable to support 3D printing because its negligible yield stress cannot support layering of material, and its shape retention is poor due to its low storage modulus. Meanwhile, the fumed silica‐based precursor ink supports 3D printing due to the enhanced yield stress and storage modulus attributed to precursor gelation. With a yield stress on the order of 300 Pa and a storage modulus on the order of 10^4^ Pa in the SAOS regime, the rheological profile of the bicontinuous emulsion precursor ink is comparable to other extrusion‐based 3D‐printable multiphasic (but non‐bicontinuous) emulsion inks.^[^ ^42^, ^43^, ^44^ ^]^

During extrusion, inks are exposed to high shear rates of ≈300 s^−1^ using the 3D printing conditions presented in this study, which we have demonstrated can alter the ink's properties, resulting in transitions from gel‐like to fluid‐like behavior before and during extrusion. However, another key factor to consider for a DIW ink's performance is its behavior after extrusion; the ink must be thixotropic such that it recovers its rheological properties to resist gravitational stresses and surface tension instabilities. Thixotropy is screened through a shear‐rate hysteresis loop, as confirmed in Figure 2c. In the hysteresis loop, stress is measured as shear rate increases during the ramp‐up curve and as shear rate decreases during the ramp‐down curve. The printable precursor supports higher stresses during the up‐curve, relative to the down‐curve. This behavior is typical of a thixotropic material, as there is a loss in apparent viscosity during high‐shear exposures. Furthermore, we observe a decrease in the measured yield stress at the beginning of the ramp‐up curve (300 Pa) and at the end of the ramp‐down curve (20 Pa). Although there is not a complete thixotropic recovery, other studies probing the relationship between ink rheology and printability find that even partial yield stress restoration across a hysteresis loop is significant when predicting the viability of DIW inks, as the ability to rebuild a yield stress after exposure to high shear rates associated with DIW extrusion is crucial.^[^ ^40^ ^]^ To further probe the post‐extrusion recovery of these rheological properties, we perform transient creep and recovery experiments. These experiments further support the thixotropic nature of the printable bicontinuous emulsion precursor; we find the precursor recovers its rheological parameters within a timescale of 40–60 s upon cessation of applied shear (see Figure S6, Supporting Information). This recovery is due to the thixotropic behavior of the precursor that is essential to prevent spreading after extrusion and helps to maintain print fidelity.

The recovery of gel‐like behavior likely plays an important role in the formation of the bicontinuous microstructure during DIW; both processes occur concurrently. After the one‐phase precursor is extruded, a two‐phase bicontinuous microstructure emerges as ethanol evaporates. Our prior work has shown that this bicontinuous morphology develops within tens of seconds.^[^ ^6^, ^45^ ^]^ This timescale is similar to the time required for gel‐like properties to be recovered in sheared fumed silica suspensions, as determined by rheological analysis (see Figure S6b–d, Supporting Information). We explore how the interplay of these two simultaneous processes influences the microstructure in the quenched bicontinuous emulsions.

### 2.2 3D Printed Bicontinuous Emulsion Gels Feature Submicron‐Sized Domains

**Figure 3:**
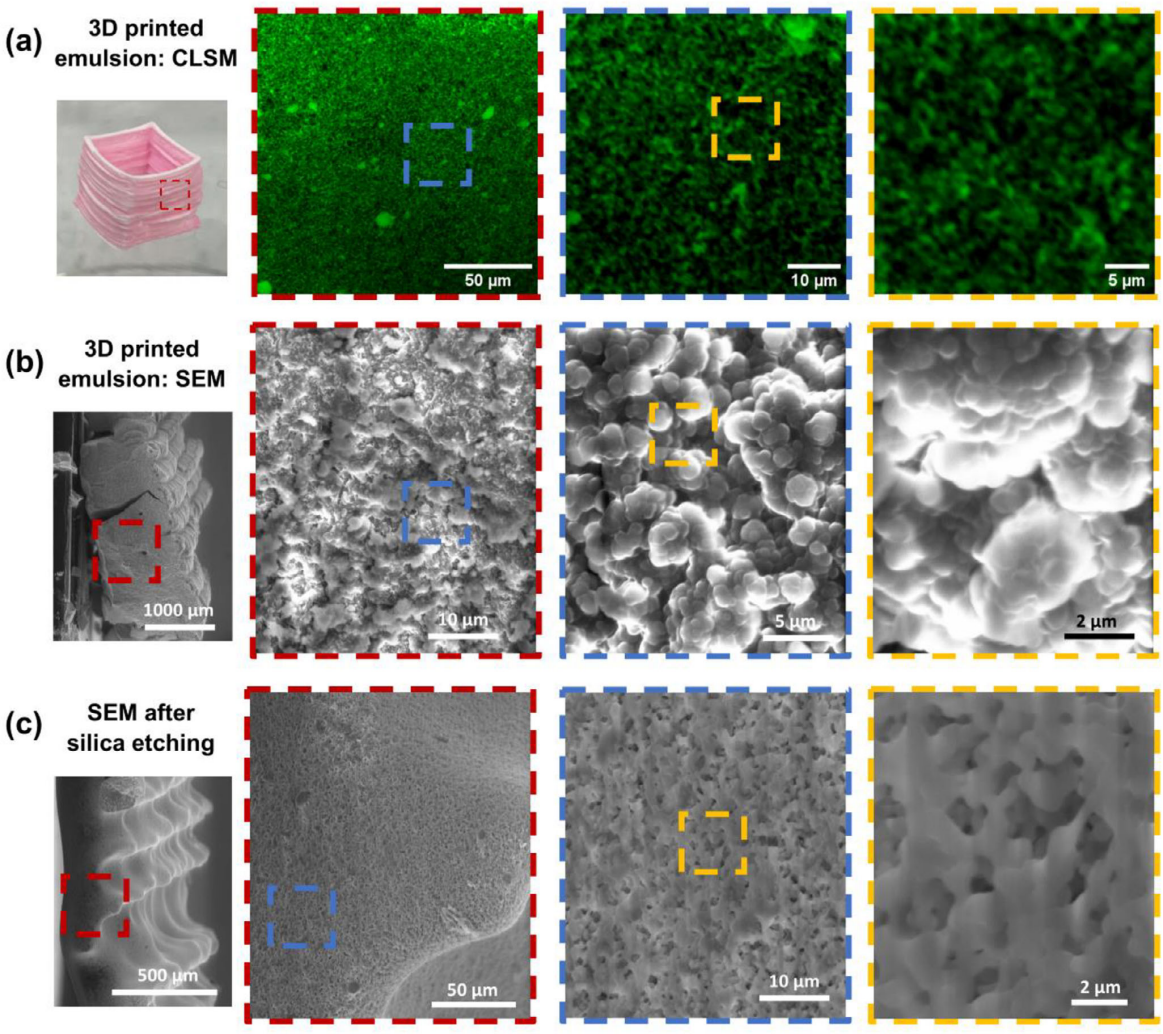
3D printed bicontinuous emulsion gels feature submicrometer‐sized domains. a) CLSM images of a section from a 3D printed bicontinuous emulsion gel. The green fluorescence signal represents polymerized oil channels and black represents pores from previously evacuated water channels. b) Cross‐sectional SEM series of a 3D printed bicontinuous emulsion gel. Polymer channels are covered with interfacially jammed fumed silica particles and excess particles. C) Cross‐sectional SEM series of a 3D printed bicontinuous emulsion gel after removal of fumed silica particles. After etching, the bicontinuous polymer‐pore microstructure is revealed.

To confirm that 3D printed structures have a bicontinuous morphology, we analyze their microstructure using CLSM and SEM. We print a 3D rectangular prism with the dimensions of 1 cm × 1 cm × 1 cm, consisting of 40 layers of filaments with a diameter of 250 µm. The structure is crosslinked under UV irradiation after 2 min of ethanol evaporation. The polymerized structure is washed in ethanol and dried in ambient conditions to remove water and any excess solvent, producing a porous polymeric scaffold. A wall is cut from the prism and viewed under CLSM. The green structures represent the fluorescent signals from the Nile red containing polymer and correspond to the oil phase of the structure. Black represents the pores formerly occupied by water. Under high magnification, the bicontinuous microstructure is revealed on length scales ranging from hundreds of nanometers to micrometers as shown in **Figure** **3**a. To have a clearer visualization of sub‐micrometer structures, we perform cross‐sectional SEM on fractured samples. Fumed silica clusters on the order of hundreds of nanometers obscure the underlying morphology as seen in Figure 3b. These particles are located at the polymer‐pore interface, indicating that they were either interfacially jammed before UV curing or were particles suspended in the aqueous phase that settled on the polymer surface after the water was removed. To reveal the porous microstructure more clearly, these silica particles are removed before SEM imaging via dissolution in a 1m NaOH solution overnight. Without silica particles, the bicontinuous and porous nature of the 3D printed material is revealed clearly as shown in Figure 3c. The sizes of the polymer and pores are on the order of hundreds of nanometers to micrometers and appear similar to the images of the structure observed under CLSM. Additionally, the surface of the printed emulsion gels is characterized via SEM (see Figure S7, Supporting Information), revealing similar structures found in the cross‐sectional analysis. We also include a 3D reconstruction of the microstructure from a fragment of a 3D printed bicontinuous gel (see Figure S8, Supporting Information).

The SEM characterization indicates the microstructures are uniformly sized throughout the 3D printed monoliths, both internally and on the surface. The structures even extend across adjacent printed layers, suggesting bicontinuity may be established across the entire structure. We confirm the bicontinuous morphology across 3D printed layers by inducing capillary infiltration across the pores of these structures (see Figure S9, Supporting Information). Demonstrating the bicontinuity of a structure experimentally is challenging. Here, we opt for a functional test to detect fluid transport across the porous material, indicating continuity of the material's porous network. We believe the observed microstructural continuity across printed layers arises from the continuous printing process. As a new precursor filament with a high concentration of ethanol is extruded onto an existing printed structure with a lower concentration of ethanol, the co‐solvent diffuses into the underlying material. This diffusion likely decreases the interfacial tension and facilitates the formation of continuous oil and water channels across layers. In contrast, structures fabricated using a “print‐cure‐print” approach, where intermediate layers are cured before additional material is deposited, fail to exhibit this continuity in microstructure and exhibit poor inter‐layer adhesion. For example, when printing a rectangular prism with 10 layers cured before depositing an additional 10 layers, the top portion cleanly separates from the bottom upon handling, indicating poor interlayer adhesion (see Video S2, Supporting Information). These results highlight the importance of ethanol‐mediated interdiffusion in preserving the bicontinuous structure throughout the printed object.

Notably, the microstructures of these 3D printed bicontinuous emulsions have features that differ from those observed in bijels prepared using spinodal‐based techniques that are based on ternary mixtures, such as VIPS or STRIPS. Bijels formed via spinodal pathways are identified through their zero mean curvature and negative Gaussian curvature along the oil‐water interface.^[^ ^18^ ^]^ Solvent transfer‐based bicontinuous emulsions typically form domain sizes on the order of tens of micrometers.^[^ ^5^, ^6^ ^]^ Recent reports have shown that submicrometer‐sized domains can be stabilized by regulating the mass transfer kinetics for solvent removal or modifying the particle surface chemistry through surfactant or covalent functionalization.^[^ ^45^, ^46^, ^47^ ^]^ Remarkably, 3D printed bicontinuous emulsions also have stable submicrometer domains, without implementing any additional regulation of co‐solvent removal dynamics or particle functionalization. This finding suggests that another structural stability‐enhancing mechanism may be playing a role that is not present in typical solvent removal‐based bicontinuous systems.

To explore this premise, we consider the complexity of the 3D printable system, as the quenched microstructure is influenced by a variety of factors. The rheology of the precursor ink implies that gel‐like structures form before the system phase separates. The two types of particles in the system both tend to form rough, fractal‐like aggregates and clusters, which likely behave differently at the interface when compared to spherical particles. Moreover, the thixotropic nature of the system indicates that gel‐like structures are likely recovered in both the aqueous and the oil phases after they are weakened during high shear extrusion.

Considering the structural difference between the 3D printed bicontinuous emulsions and the conventional VIPS‐based system,^[^ ^6^ ^]^ it is possible that these 3D printed microstructures form through a non‐spinodal pathway. Bicontinuous emulsions fabricated through non‐spinodal paths have been previously reported. For example, VIPS bijels were formed through a partial coalescence mechanism, where a percolating network forms through nucleation and growth of many droplets that bridge together due to incomplete interfacial particle assembly.^[^ ^6^ ^]^ Further, bijels and bigels can be formed via direct mixing of two immiscible fluids.^[^ ^7^, ^9^ ^]^ As such, spinodal decomposition is not a requirement for bicontinuous emulsions production. Given the lack of distinct spinodal‐like structures in the 3D printed bijel system, despite optimizing the ternary liquid mixture around its critical point, questions arise surrounding the stabilization mechanism of these VIPS generated structures. In particular, the relative importance of interfacial jamming or other mechanisms such as bulk stabilization is worthy of discussion.

The interfacial activity of the fumed silica particles, as shown in Figure S1 (Supporting Information), suggests that the 3D printed bicontinuous emulsions are stabilized, at least in part, through interfacial attachment and jamming of adsorbed particles. Furthermore, the high fraction of particles present in the system, and the complex rheology of these suspensions indicate that particles also form structures within both the HDA and water phases. Upon extrusion, bulk phase gelation is diminished, as shown in Figure 2; during this reduced gelation regime, fumed silica can undergo rearrangements and sample the local environment via diffusion. This freedom may promote particle reorientation and adsorption to the HDA/water interface as phase separation generates the interface with large enough surface tension to trap particles. Further, during this quenching process, co‐solvent is continuously removed from the system, increasing the interfacial tension, further promoting particle trapping with larger attachment energies (see Figure S10, Supporting Information). Given that gel recovery and VIPS‐driven phase separation occur on similar timescales of tens of seconds, particles dispersed in both the HDA and water phases likely form percolating networks within each bicontinuous domain. These gelation processes help suppress coarsening and coalescence, allowing for stabilization of bicontinuous emulsions with submicrometer‐sized domains. Operating in tandem, the two mechanisms of interfacial jamming and bulk phase gelation have been shown to synergistically enhance the elasticity of Pickering emulsions.^[^ ^48^ ^]^ We believe that both mechanisms also contribute to the stabilization of the submicrometer‐sized bicontinuous emulsion gels.

### 2.3 DIW Allows for Emulsion Fabrication with Complex Geometries

**Figure 4:**
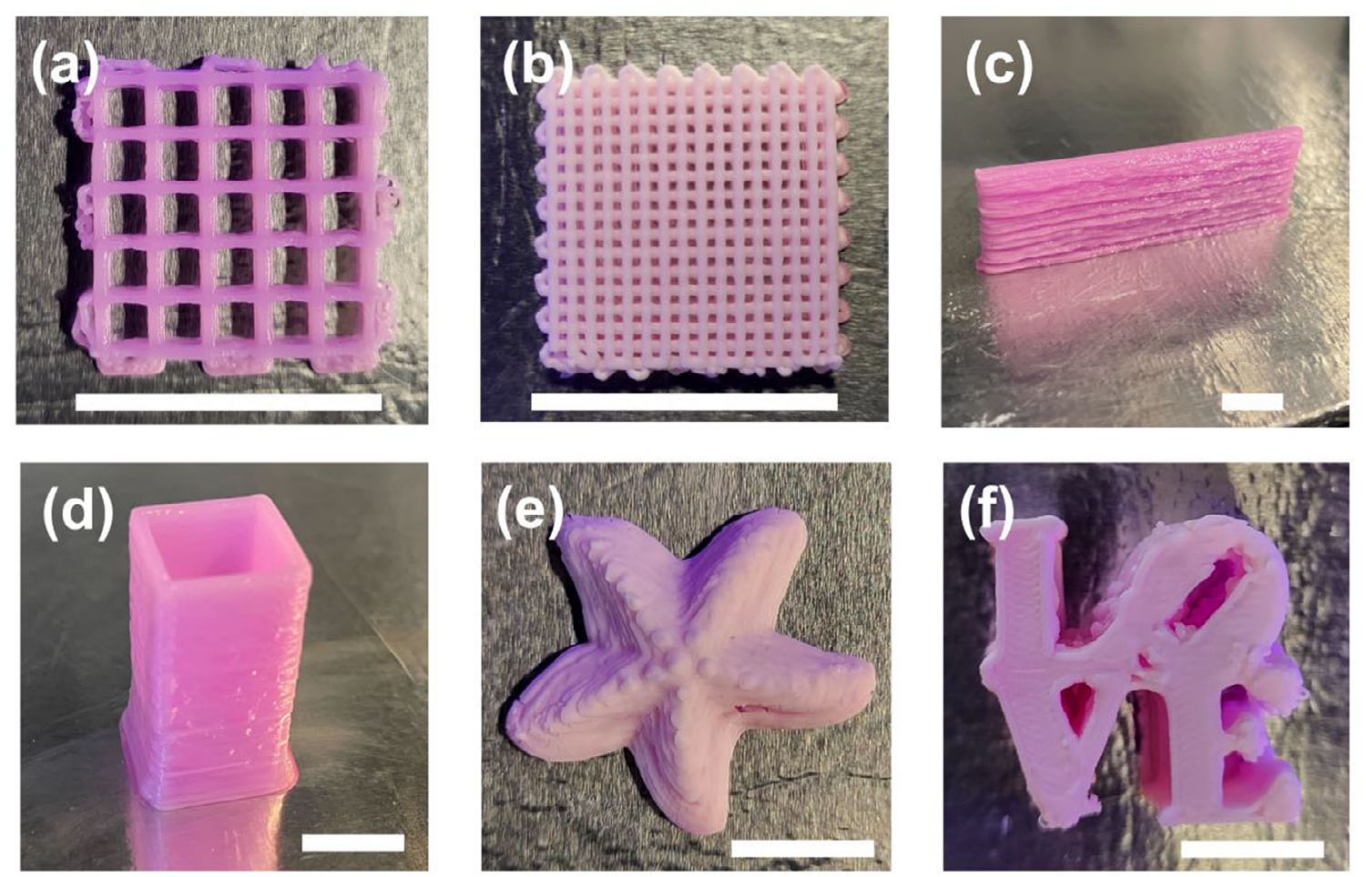
DIW allows for emulsion gel fabrication with complex geometries. Bicontinuous emulsion gels printed with macroscale morphologies of: a) high span woodpile grid, b) low span woodpile grid, c) vertical wall, d) vertical rectangular prism, e) starfish, and f) the Philadelphia LOVE sculpture. All scale bars represent 1 cm.

Using our bicontinuous emulsion gel precursor ink, we print designs of varying complexity, such as a starfish and Philadelphia LOVE sculpture, using a 250 µm nozzle at a printing speed of 15 mm s^−1^ as shown in **Figure** **4** (see Videos S3 and S4, Supporting Information). Print fidelity is influenced by ink properties, such as yield stress and surface adhesion, and the printing parameters, such as extrusion pressure, nozzle diameter, and print speed. We optimize printing parameters to prevent print failure; if filament gaps are formed (under‐extrusion), the extrusion pressure is increased or the print speed is decreased. If the filament buckles or swells (over‐extrusion), the extrusion pressure is decreased or the print speed is increased. We explore how the print speed, printing pressure, and nozzle size influence the filament behavior in Figure S11 (Supporting Information). The structures demonstrated in Figure 4 are printed with a 250‐micrometer inner diameter tapered nozzle. Larger diameter nozzles can be used, but this may reduce the 3D printing precision, as the printing resolution is typically limited to the smallest nozzle diameter compatible with the ink for DIW‐based printing. We note that printing the bicontinuous emulsion precursor with nozzles smaller than 250‐micrometers gives inconsistent results, as smaller nozzles are more prone to clogging. Additionally, opaque barrels and nozzle tips are used to prevent nozzle clogging due to premature ambient UV polymerization. Further, the mechanical response of the cured bicontinuous emulsions under compression reveals brittle behavior (see Figure S12, Supporting Information). We believe reinforcement of the bicontinuous morphology can improve these properties, providing robustness in applications with strict load‐bearing requirements. Additionally, the mass and dimensions of printed structures tend to decrease across the quenching and curing processes, due to the presence of volatile co‐solvent in the precursor, whose evaporation is necessary to drive phase separation during the quenching process (see Figure S13, Supporting Information).

Our results clearly demonstrate that this 3D printing technique can be used to produce bicontinuous emulsions with complex, hierarchical geometries in a scalable manner. This is particularly useful when creating porous materials with sub‐micrometer domains, which are of interest for electrodes for energy storage, membrane separations, bioscaffolding, bioengineering, and other domains. Further, we anticipate that print fidelity can be improved further with more advanced DIW configurations including implementation of layer‐by‐layer or partial UV curing, employing sacrificial scaffolding materials, or through humidity control of the printing chamber.

## 3 Conclusion

We have developed a method to 3D print bicontinuous emulsion gels using DIW. By 3D printing these nanoparticle‐stabilized structures, this technique allows for the continuous fabrication of hierarchical porous materials with complex and user‐customizable geometries. We realize 3D printing by introducing mixtures of hydrophilic and hydrophobic fumed silica into the ternary liquid oil/water/co‐solvent emulsion gel precursor. Fumed silica particles induce particle gelation in the precursor, significantly altering the precursor's rheological profile to meet requirements for DIW. The rheological characterization of the precursor reveals a sufficient yield stress and strong storage modulus in the low stress LVR, supporting material layering during extrusion and promoting shape retention after extrusion. Once the precursor filament is extruded from the nozzle, bicontinuous emulsion formation progresses through ambient vaporization‐induced phase separation. By UV curing the printed emulsion and drying to remove excess liquid, we create a hierarchical porous material. Characterization via CLSM and SEM imaging shows that the 3D printed bicontinuous emulsions possess sub‐micrometer domains. We propose that these emulsions are stabilized by two mechanisms that occur simultaneously. The DIW ink, whose network is weakened by high shear during printing, allows particle clusters to adsorb and jam along the interface during phase separation. At the same time, particle clusters in each domain re‐form gels and stabilize the bicontinuous morphology via the formation of percolating phase‐spanning networks. These findings are supported by comparison of the thixotropic gel recovery timescale and the VIPS bijel formation timescale, which are both on the order of tens of seconds. These mechanisms are enhanced by volume reduction effects as ethanol evaporates from the system, further increasing surface tension and particle concentration, providing greater jamming along the interface and gelling within each domain, respectively. We demonstrate DIW capabilities through printing 3D structures of various levels of complexity, creating a hierarchical, biphasic, particle‐stabilized, and surfactant‐free material. These 3D printed bicontinuous materials offer unique advantages for applications in reactive separation, tissue engineering, and membrane separation. Their bicontinuous morphology promotes bulk transport within each phase, while the large interfacial area between two immiscible phases enables efficient exchange across the interfaces. These materials also exhibit distinctive optical properties, which have been exploited in radiative coatings. Importantly, 3D printing enables the fabrication of bicontinuous structures with tunable mechanical properties. Functionalized bicontinuous emulsions can serve as scaffolds for cell growth and delivery, biomedical implants, filtration and separation devices, and electrodes for energy storage.

## 4 Experimental Section

### Materials

The emulsion precursor ternary mixture consists of 1,6‐hexanediol diacrylate (HDA, 99%, Sigma), water, ethanol (EtOH, 200 proof, Decon Laboratories, Inc.). The water in the ternary mixture was adjusted to a pH of 3 using a 1 m solution of HCl (Fisher Chemical). Hydrophilic fumed silica particles (CAB‐O‐SIL LM‐150) and hydrophobic fumed silica particles (CAB‐O‐SIL TS‐610) were provided by Cabot Corporation. For UV curing polymerization, 2‐hydroxy 2‐methylpropiophenone (HMP, 97%, Sigma) was added into the mixture. For fluorescence imaging via CLSM, Nile Red (microscopy grade, Sigma) and 9,10‐bis(phenylethynyl)anthracene (BPA, 97%, Sigma) were used as fluorophores to image the two emulsion phases. For permeation experiments, polymerized emulsions are submersed in diethyl phthalate (DEP, 99.5%, Sigma).

### Bicontinuous Emulsion Gel Precursor Ink Preparation

The components of the 3D printable precursor are: 1) HDA, 2) water, 3) ethanol, 4) hydrophilic fumed silica, 5) hydrophobic fumed silica, and 6) HMP. To form a typical printable precursor, these components were mixed with the following ratios of components and in the listed order: 1) 3.82 g, 2) 4.28 g, 3) 5.79 g, 4) 1.60 g, 5) 0.72 g, and 6) 0.35 g. The mixture was shaken after ethanol was added to the mixture to form a miscible ternary solution. After the hydrophilic fumed silica particles were added, the mixture was shaken and vortexed until all visually large agglomerates of silica were dispersed. Then the hydrophobic fumed silica particles were added to the mixture and the mixture was vortexed and then sonicated for 45–60 min. The mixture was stored in the dark to prevent any ambient UV polymerization. The precursor is sonicated for 45–60 min before each experiment to reduce aggregation of silica particles.

### 3D Printing Protocol

Prior to printing, the precursor ink was transferred to an opaque black 10cc syringe barrel (Nordson). The barrel was fitted with a 25G opaque rigid dispensing tip (Nordson) with an inner diameter of 250 µm. Opaque components were selected to prevent premature polymerization of the bicontinuous emulsion due to ambient UV exposure, which would obstruct the nozzle tips and inhibit extrusion. Samples are printed using a MakerGear M2 3D Printer modified with a pneumatic control apparatus for compatibility with a wide range of inks at room temperature. The build plate was a MakerGear borosilicate glass build surface coated with Bytac, a laminate composed of a fluorinated ethylene propylene film bound to an aluminum sheet. Printing speed and applied pressure were adjusted until the ink maintains filament fidelity. After extrusion, the printed structures undergo polymerization via UV irradiation (320–500 nm, 40 W cm^−2^). To demonstrate printability, 1 cm × 1 cm woodpile‐structured scaffolds with span factors of 1 and 4 were printed, as well as a 75‐layer wall, a 100‐layer hollow rectangular prism, a 1:100 replica of the Philadelphia LOVE sculpture, and a starfish.

### Rheological Characterization

A TA Instrument DHR‐3 rheometer was employed with a 40 mm cone‐plate geometry. A solvent trap was installed to minimize the effects of ethanol evaporation on rheology measurements of the emulsion precursor. For all measurements, temperature control is implemented and set to 25 °C. For flow‐sweep experiments, stress and viscosity are measured at shear rates of 0.01, 0.1, 1.0, and 10 s^−1^. The stress versus shear rate data is fit to the Hershel–Bulkley model to determine the yield stress. For oscillatory amplitude‐sweep experiments, the storage (G′) and loss (G″) moduli are measured for increasing amplitudes, with a constant frequency of 2π rad s^−1^. To assess thixotropy of the precursor, viscosity is measured transiently, with stepwise shear rates of 0.01, then 300, then 0.01 s^−1^. Thixotropy was also tested via hysteresis loop, where stress was measured as shear rate increases then subsequently decreases.

### Confocal Laser Scanning Microscopy Characterization

To enable fluorescence imaging of emulsion structures, a trace amount of Nile red was mixed into the precursor after all components were mixed. Nile red is a hydrophobic fluorophore and selectively partitions into the emulsion's oil phase during phase separation via ethanol evaporation. After UV‐induced polymerization of the HDA phase and drying of the water phase, Nile red remains inside of the polymer phase, and as a result, an Olympus FV1000 confocal laser scanning microscope can be used to image the polymerized emulsions. Polymerized emulsions were immersed in DEP, which permeates into the pores of the polymerized emulsion. To image the pore phase, trace amount of BPA is added to DEP, which is a second fluorophore. The polymerized emulsion was immersed in DEP for at least 10 min before imaging under confocal microscopy. A 488 nm laser was chosen for fluorescence excitation. Two separate emission channels were monitored to image the polymerized emulsion: 490–520 nm to image the pore phase (BPA emission) and 560–660 nm to image the polymer phase (Nile red emission). Each image was acquired at 512 × 512 pixels with a scan rate of 200 µs per pixel. The images were processed in ImageJ for false coloring.

### Scanning Electron Microscopy Characterization

A FEI Quanta 600 FEG Mark II SEM was employed for imaging of 3D printed bicontinuous emulsions after UV curing and drying. The instrument was operated under high vacuum at a pressure of 0.38 torr. Images were acquired by applying a 5–10 kV electron beam along with a spot size of 3.0. Images were taken across a length of magnifications, ranging from 50x to 70000x. 4 nm Ir was sputter‐coated on to the viewing surface, to reduce surface charging from silica particles.

## Conflict of Interest

The authors declare no conflict of interest.

## Supporting information



Supporting Information

Supplemental Video 1

Supplemental Video 2

Supplemental Video 3

Supplemental Video 4

## Data Availability

The data that support the findings of this study are available from the corresponding author upon reasonable request.
