# Supplementary material for: 3D Printing of Bicontinuous Nanoparticle‐Stabilized Emulsion Gels via Co‐Solvent Removal
Source: Small. 2025 Nov 3;21(51):e04718. doi: 10.1002/smll.202504718 (PMC12723326; doi:10.1002/smll.202504718)
Supplement: Supplementary file 1 — Supporting Information [file SMLL-21-e04718-s002.docx]

Supporting Information

**3D Printing of Bicontinuous Nanoparticle-Stabilized Emulsion Gels via Co-Solvent Removal**

*Philip R. Iaccarino, Damilola Lawal, Jordan R. Raney, Kathleen J. Stebe*, and Daeyeon Lee**

**Interfacial activity of fumed silica particle mixture**

We test the ability of the two fumed silica particles to assemble and stabilize HDA/water interfaces. We form oil-in-water emulsions of HDA droplets in water by mixing at 10,000 rpm for two minutes, with and without fumed silica (FS). If the two particles are present in the mixture, the emulsion remains stable for at least a week, whereas the emulsion destabilizes rapidly with evidence of macroscale phase separation within 120 minutes if particles are not present, as seen in Figure S1a. This implies the presence particles at the interface. These HDA droplets are polymerized via UV curing to allow for SEM imaging in Figure S1b, where clusters of fumed silica are seen on the surface of these polymerized droplets further supporting the interfacial assembly of the two fumed silica particles.


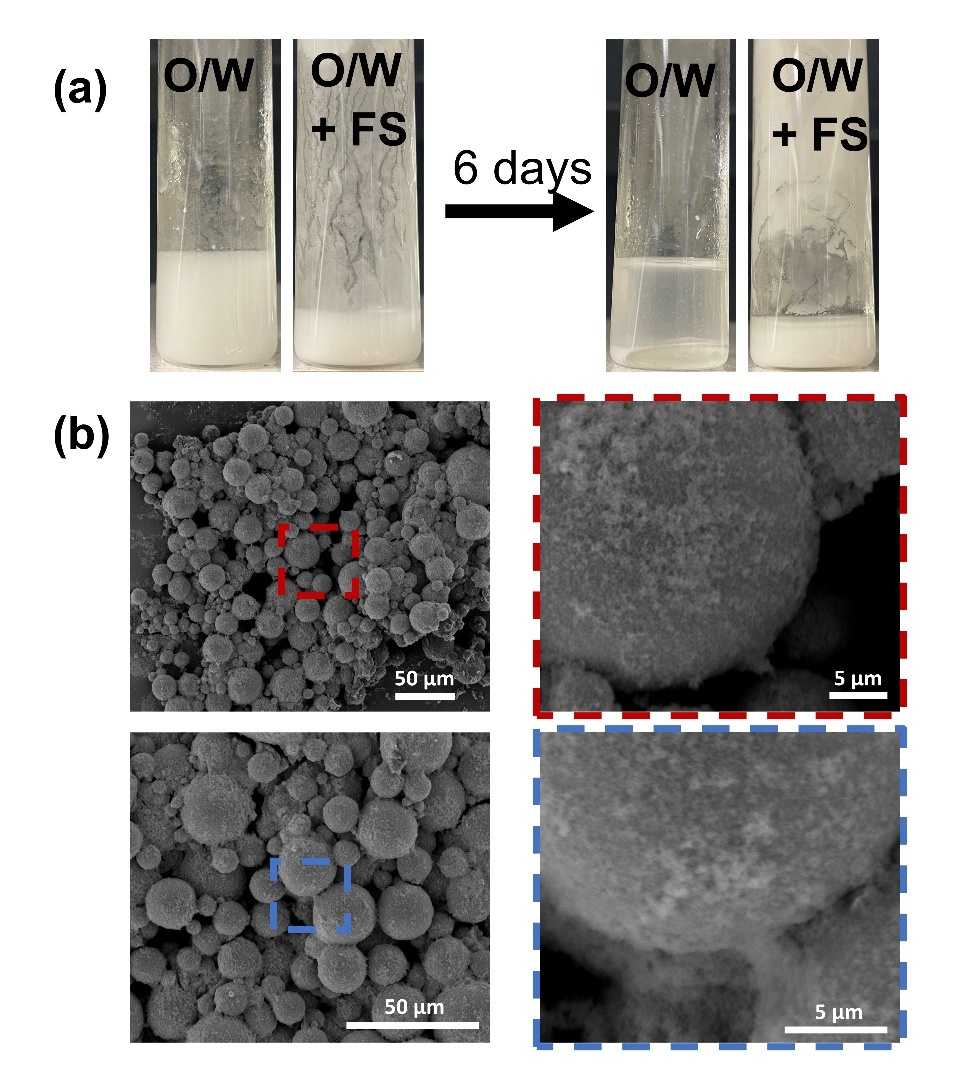


**Figure S1.** Interfacial activity of fumed silica particle mixture. a) Emulsions of water and HDA are mixed, with and without FS particles. When FS particles are present, the emulsion remains stable for at least 7 days, as indicated by turbid and cloudy liquid appearance. When particles are not present, the oil-in-water demixes during the 7-day period, as transparency is restored. b) SEM series of polymerized oil droplets containing FS particles. The particles are observed at the interface, showcasing their ability to stabilize HDA/water interfaces.

**Dispersibility of fumed silica particles**

The fumed silica particles are used as received by the manufacturer. They are classified as “hydrophobic” and “hydrophilic” but their ability to be dispersed in HDA, water, and ethanol was unknown, a priori. To confirm their dispersibility in these solvents, low concentration mixtures of particles are formed. We prepare mixtures the hydrophilic and hydrophobic particles at 2 wt% in each of the three solvents, and their dispersibility is assessed via inspection in Figure S2. Both particle variants can be dispersed in HDA and in ethanol. In water, the hydrophobic variant cannot be dispersed whereas the hydrophilic variant can be. Fumed silica typically has a refractive index of 1.46 which is essentially the same as the index of refraction of HDA at 1.46; therefore, the dispersion is transparent. Water and ethanol have an index of refraction is 1.33 and 1.36 respectively, and thus the ternary mixture is expected to have the refractive index of 1.37 (based on the volume fraction of the three liquids); as a result, the dispersions are cloudy and opaque.


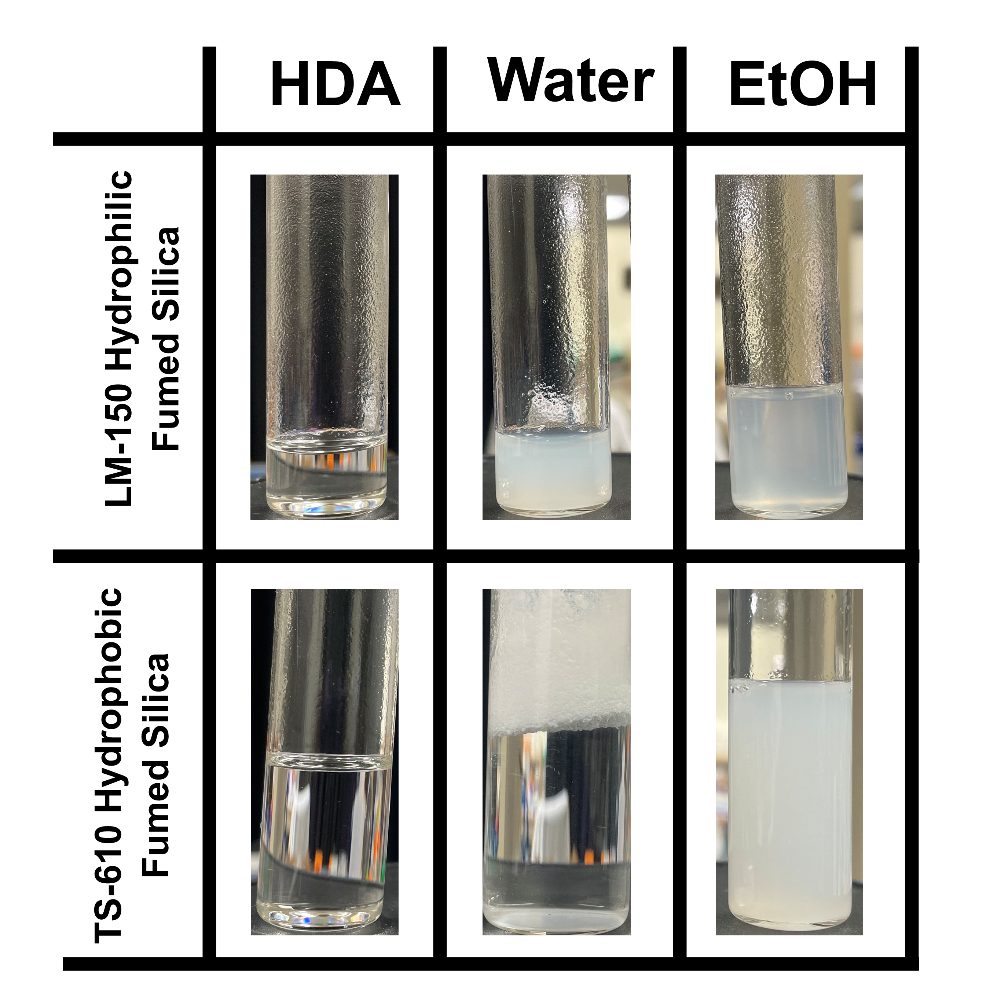


**Figure S2.** Dispersibility of fumed silica particles**.** The hydrophilic and hydrophobic fumed silica particles are individually mixed into HDA, water, and ethanol, separately, to assess their dispersion behavior in each solvent. Hydrophilic LM-150 fumed silica particles can be dispersed in all diluents, while hydrophobic TS-610 fumed silica particles can only be dispersed in HDA and ethanol.

**Ternary phase diagram construction and critical point determination**

The precursor mixture is designed such that its ternary liquid composition is near the critical point in the ternary phase diagram to facilitate formation of bicontinuous fluids upon phase separation via VIPS. The critical point is found by comparing quenched emulsion structures from precursors with compositions just outside the binodal regime. However, to do this, the binodal line must be constructed. The binodal line is mapped by measuring the amount of ethanol required to mix blends of HDA and water into a one-phase system. Equal amounts (1.00 g) of water and HDA are added to a glass vial. Ethanol is added dropwise into the vial while gently stirring, and the phase behavior is observed visually. If the mixture appears turbid and cloudy, the system is in an immiscible two-phase state. When enough ethanol is added, the system transitions to an optically transparent, miscible one-phase state. The amount of added ethanol to produce this turbid-to-transparent transition is recorded to tabulate the first data point of the binodal curve. Then, the amount of water in the solution is increased by ~5% to create a turbid immiscible two-phase system and this added water mass is recorded. Ethanol is added dropwise again until the mixture becomes transparent. Considering the total mass of water, HDA, and ethanol in the mixture, a second data point of the binary curve is tabulated. This process is repeated until the turbid to transparent transition can no longer be observed visually, resulting in half of the binodal curve. To produce the other half of the curve, the whole process is repeated starting from equal volumes of HDA and water, but now ~5% of HDA is incrementally added for each data point, rather than water. The two data sets are joined to plot the experimentally determined binodal line of the HDA-water-ethanol ternary phase diagram. The corresponding ternary phase diagram is shown in Figure S3a.

**Table S1**. Composition of precursors A, B, C, D along binodal line in ternary phase diagram.

| Component | A | B | C | D |
| --- | --- | --- | --- | --- |
| HDA | 14.3 vol% | 19.8 vol% | 25.1 vol% | 30.3 vol% |
| Water | 33.8 vol% | 31.9 vol% | 28.5 vol% | 24.8 vol% |
| Ethanol | 51.8 vol% | 48.3 vol% | 46.4 vol% | 44.8 vol% |
| LM-150 | 9.04 wt% | 8.62 wt% | 8.66 wt% | 8.68 wt% |
| TS-610 | 2.45 wt% | 2.21 wt% | 2.31 wt% | 2.40 wt% |
| HMP | 1.57 wt% | 2.04 wt% | 2.45 wt% | 3.04 wt% |


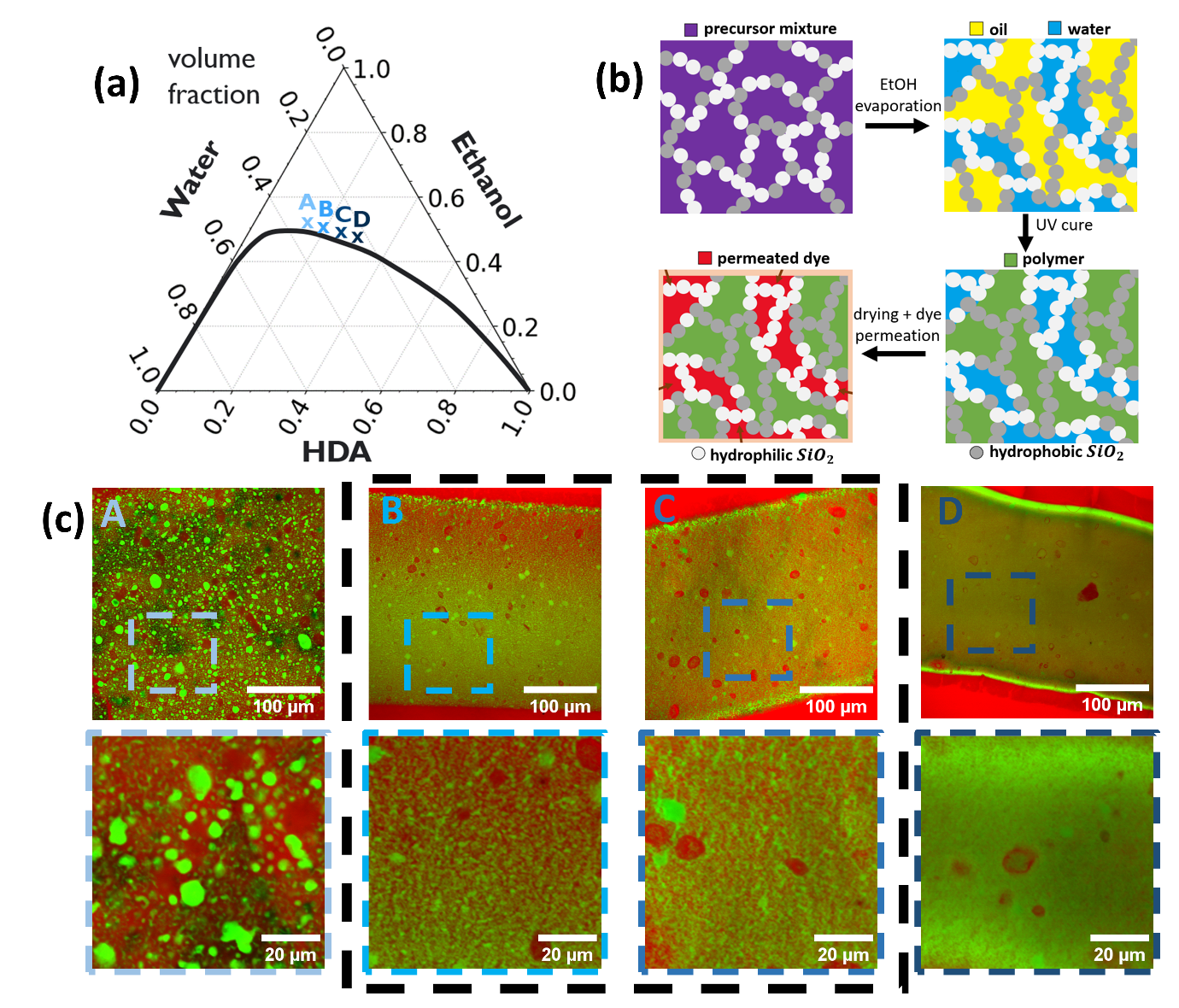


**Figure S3.** Ternary phase diagram construction and critical point determination. a) Ternary phase diagram of HDA, water, and ethanol. Precursors with compositions from points A through D in the miscible one-phase region are prepared, then quenched into the immiscible two-phase region via ambient evaporation of ethanol. b) Schematics describing CLSM sample preparation process. c) CLSM of precursors A through D after quenching and polymerization reveals a transition from oil-in-water emulsions (A) to water-in-oil emulsions (D) as the oil concentration increases, with bicontinuous structures formed near the transition critical point (B and C).

Structures of quenched emulsions with varying compositions are compared to determine the critical point along the binodal line. Four precursors (A, B, C, D) are prepared, with increasing ratios of oil-to-water; their compositions are listed in Table S1. The precursors are formulated such that the ternary liquid components (HDA, water, ethanol) consist of the volume components and are expressed as volume percentages, while the particles are expressed as weight percentages relative to the entire mixture. To simplify the formulation processes, we assume the particles do not influence the phase boundaries in the ternary phase diagram. Ethanol is added to create a miscible one-phase precursor, such that the composition is located above the binodal line in Figure S3a. The precursors are extruded onto a glass slide, then quenched via ambient ethanol evaporation for one minute, followed by UV curing to form polymer-pore networks. With trace amount of hydrophobic fluorescent dyes (Nile red and BPA) introduced into the precursor and immersion fluid, we image the polymerized emulsions via CLSM as per the procedure in Figure S3b, where green signal represents the polymerized oil phase and the red signal represents the permeated pore phase water that was formerly occupied by water. As seen in Figure S3c, at a low oil-to-water ratios (composition A), oil-in-water emulsion structures are found. Similarly, at high oil-to-water ratios (composition D), water-in-oil emulsions form. Between the two, we observe a transition regime (compositions B and C), where bicontinuous emulsions form, indicating the system quenching into the spinodal region.

**Fumed silica wetting behavior and pore phase permeation**

The effect of the ratio of hydrophilic-to-hydrophobic fumed silica is explored. To do so, precursors are prepared with hydrophilic ratios ranging from zero to one, where the hydrophilic ratio is calculated as the mass ratio of hydrophilic fumed silica particles to total fumed silica particles. As such, a hydrophilic ratio of zero implies all particles in the system are the hydrophobic variant, while a hydrophilic ratio of one implies all particles in the system are the hydrophilic variant. These precursors are quenched via solvent evaporation and subsequently UV cured, and their structure is analyzed via CLSM.

For low hydrophilic ratios, water-in-oil emulsions are produced, as demonstrated in Figure S4a for a hydrophilic ratio of 0.4. On the other hand, oil-in-water emulsions are found for high hydrophilic ratios, such as a value of 1.0 in Figure S4c. For intermediate values, the fumed silica clusters can stabilize bicontinuous emulsions, as seen in Figure S4b with a hydrophilic ratio of 0.7. We identify a neutrally wetting region where bicontinuous emulsions are stabilized for hydrophilic ratios between 0.6 and 0.8, agreeing with the previously reported value where fumed silica-based bijels were formed via STRIPS.^[1]^


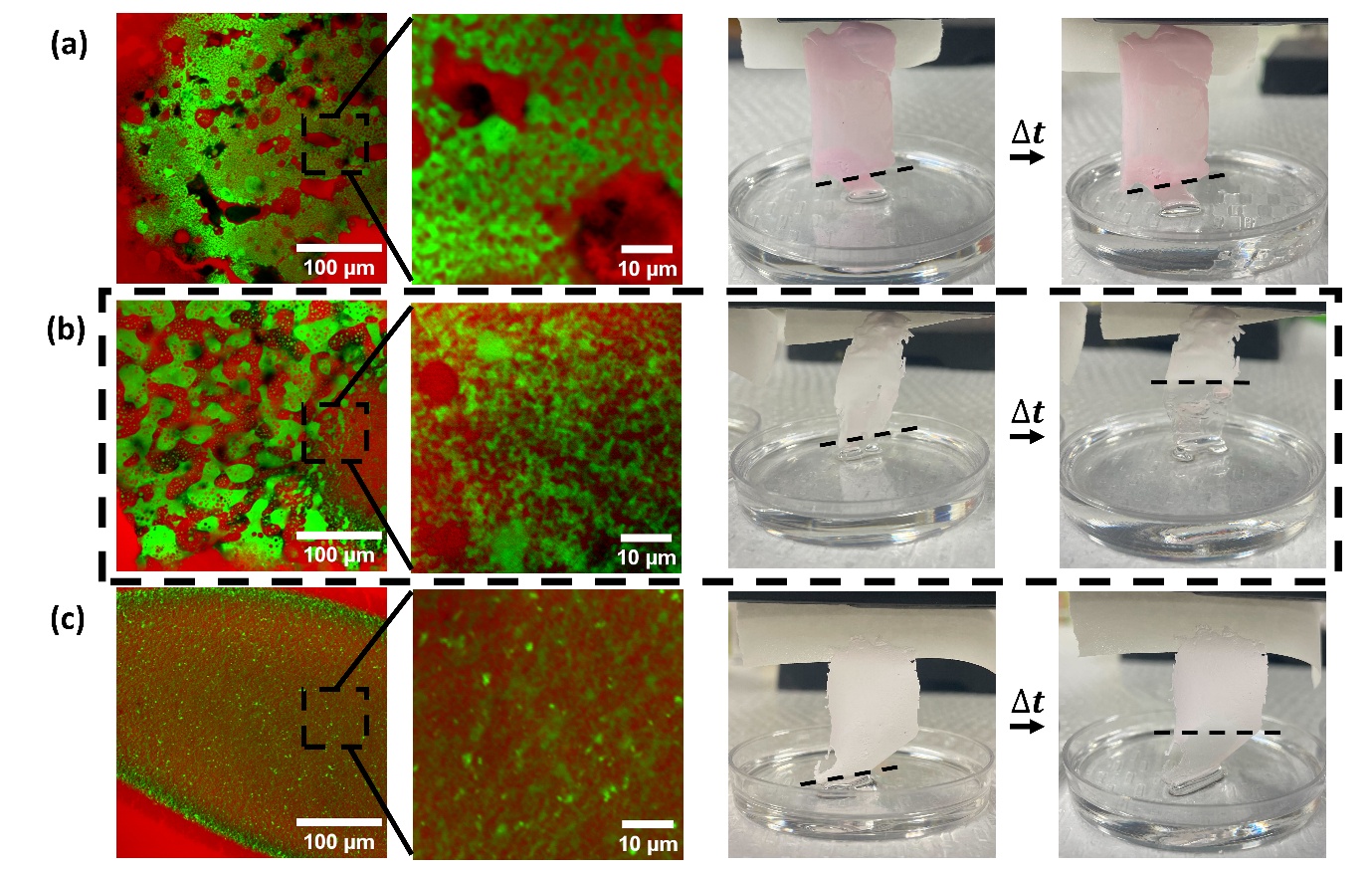


**Figure S4.** Fumed silica wetting behavior and pore phase permeation. a) At low hydrophilic ratios (0.4), CLSM reveals water-in-oil emulsion structures and DEP is unable to permeate across the discrete pores. b) For moderate hydrophilic ratios (0.7), CLSM reveals bicontinuous emulsion structures and DEP permeates across the continuous pores. c) At high hydrophilic ratios (1.0), CLSM reveals oil-in-water emulsion structures and DEP weakly permeates across the continuous pores.

Further, pore continuity is explored via capillary driven permeation. Since DEP has a similar index of refraction as poly(HDA), we can visually monitor its flow within the pores of a dried, polymerized emulsion film. We suspend the dried films in the air and dip one end into a pool of DEP. As DEP infiltrates the film’s pores via capillarity, the film transitions from a visually opaque to a visually transparent state; the behavior of the moving DEP front provides insight into the continuity of the pore phase. If there is no rise of DEP in the film, then the pores are classified as discrete which is consistent with water-in-oil emulsion behavior, as seen in Figure S4a in the low hydrophilic ratio scenario. If there is a rise of DEP, like in Figure S4b and S4c, then there is some degree of pore phase continuity; we note this phenomenon is more prominent in the intermediate hydrophilic ratio scenario, as the height of the DEP front is higher than the high hydrophilic ratio scenario. This occurs since the pore sizes are smaller when bicontinuous emulsions are stabilized for intermediate hydrophilic ratio, producing stronger capillarity. For high hydrophilic ratio, the pores are still continuous but are larger and therefore capillarity is weaker; this behavior is consistent with oil-in-water structure.

**Size determination of fumed silica clusters via dynamic light scattering (DLS)**

A DelsaNano C is used to measure the size of fumed silica clusters in each of the following diluents: water, HDA, and ethanol. The size is calculated as a Stokes-Einstein spherical particle. However, fumed silica nanoparticles have fractal-like structures; therefore, DLS gives “effective hydrodynamic diameter”. Measurements are taken at 25 °C for each experiment. The following viscosity values are used: 0.8904 cP for water, 9 cP for HDA, and 1.102 cP for ethanol. The refractive index values are 1.33 for water, 1.46 for HDA, and 1.36 for ethanol. Dispersions of LM-150 and TS-610 particles in water, HDA, and ethanol are prepared at a concentration of 2 wt%. Additional particles are added to some dispersions to increase signal intensity. The intensity distribution is collected for each dispersion and shown in Figure S5. The effective hydrodynamic diameter of all fumed silica dispersions is hundreds of nanometers.


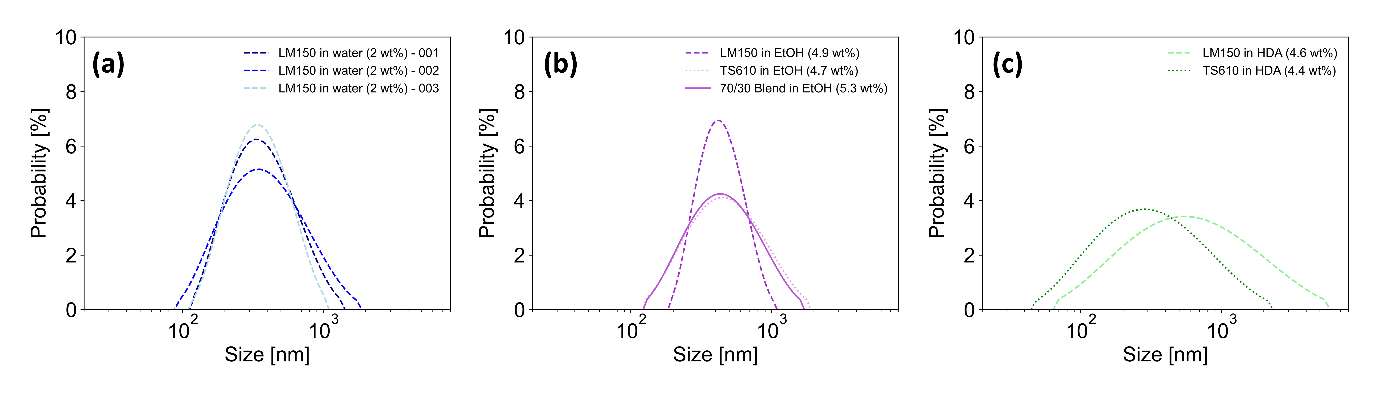


**Figure S5.** DLS size measurements of fumed silica clusters in different diluents. The peak size of the fumed silica clusters are on the order of hundreds of nanometers. a) Size distribution of hydrophilic LM-150 particles dispersed in water (three data sets). b) Size distributions of LM-150, TS-610 and a blend of particles dispersed in ethanol. c) Size distributions of LM-150 and TS-610 dispersed in HDA.

**Hershel-Bulkley fluid parameters**

The flow sweep stress vs. shear rate rheological data is fit to a Hershel-Bulkley fluid model, as defined by the equation $\sigma=\sigma_{y}+k\dot{\gamma}^{n}$ where $\sigma$ is the shear stress, $\sigma_{y}$ is the yield stress, $k$ is the consistency factor, $\dot{\gamma}$ is the shear rate, and $n$ is the power-law flow index. The Hershel-Bulkley fluid parameters are summarized in Table S1, as the concentration of fumed silica in the precursor is increased. Further, we also note if the precursor is printable via DIW extrusion for that given concentration of fumed silica.

**Table S2**. Hershel-Bulkley parameters from fitting stress vs. shear rate data for bicontinuous emulsion gel precursors as the fumed silica particle concentration is increased.

| SiO_2_ content [wt%] | Yield stress $\boldsymbol{\sigma}_{\boldsymbol{y}}$ [Pa] | $\boldsymbol{k}$ [Pa s^n^] | $\boldsymbol{n}$ | Printable? |
| --- | --- | --- | --- | --- |
| 12.6 | 367 ± 18 | 119 ± 27 | 0.527 ± 0.085 | Yes |
| 11.9 | 24.0 ± 5.1 | 108 ± 6 | 0.273 ± 0.015 | No |
| 10.6 | 18.7 ± 4.6 | 19.5 ± 7.6 | 0.802 ± 0.159 | No |
| 10.0 | 4.33 ± 0.38 | 12.2 ± 0.6 | 0.564 ± 0.018 | No |

**Rheological viscoelastic properties of the printable precursor**

Before extrusion, the bicontinuous emulsion precursor exhibits gel-like behavior, demonstrated in Figure S6a through small amplitude oscillatory shear (SAOS). Here, the storage modulus (G’) is an order of magnitude larger than the loss modulus (G’’), where both values are independent of the ink’s stress, which is described as a linear viscoelastic plateau (LVR). These factors are characteristic of gels appropriate for DIW applications.


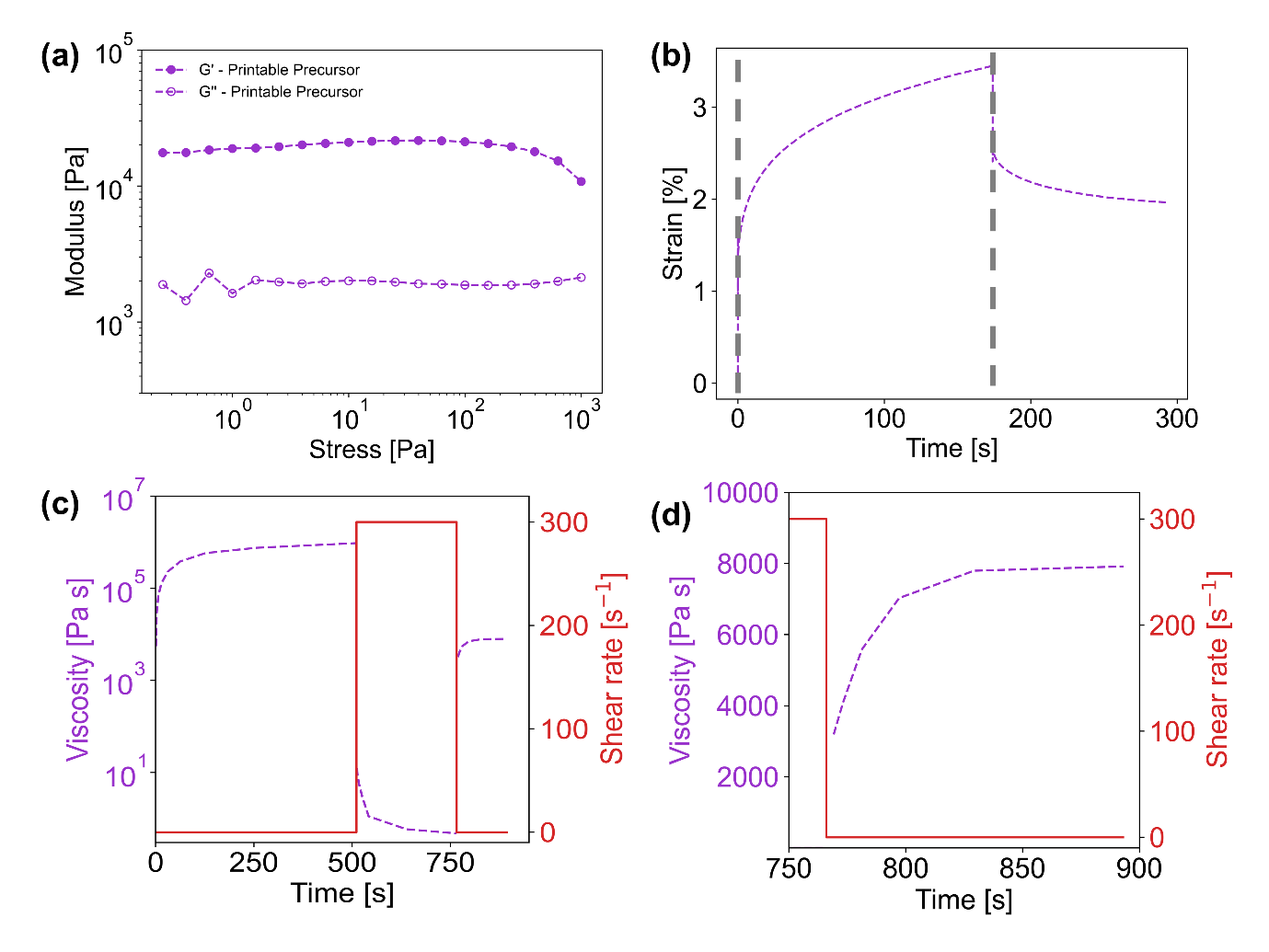


**Figure S6**. Rheological viscoelastic properties of the printable precursor. a) SAOS reveals gel-like behavior of the printable precursor in low stress environments, with a characteristic linear viscoelastic plateau of the storage modulus. b) Creep experiments show the relaxation of the viscoelastic precursor after removal of the applied stress with a timescale of 40-60 seconds. c) The transient stepwise shear-rate experiment simulates DIW extrusion; the first step simulates in-barrel motion as the precursor experiences a small shear-rate, after which the shear-rate increases to a large value to simulate a high shear extrusion environment, and lastly the shear-rate is removed to simulate the post-extrusion environment. d) The final step of the transient stepwise experiment is replotted to observe and measure the gelation timescale, as the particle gel reforms within 40-60 seconds after DIW extrusion.

The post-extrusion behavior is analyzed through a series of transient experiments. We perform creep and recovery experiments in which a 100 Pa stress is loaded onto the printable precursor for three minutes and subsequently removed. During this time, the evolution of the material’s strain is measured, as shown in Figure S6b. When stress is loaded onto the material, the precursor responds with an increase in strain. Upon removal of the stress, the precursor partially relaxes, reaching a plateau around 2% strain within 60 seconds, corresponding to recovery of the gel-like state.

Further, we characterize the three-step transient thixotropic response of the precursor to simulate conditions experienced during 3D printing, as shown in Figure S6c. In the first step, the printable precursor is exposed to a low shear rate of 0.01 s^-1^ to simulate “in-barrel” behavior, as if the precursor is slowly moving down the wider portion of the printer’s syringe before extrusion. In this regime, the precursor exhibits a high apparent viscosity, as the applied shear rate is insufficient to disrupt the precursor’s elastic gel network. Thereafter, the shear rate is rapidly increased to 300 s^-1^, to simulate conditions experienced by the material as it is extruded through the deposition nozzle. Lastly, the shear rate is rapidly decreased to 0.01 s^-1^ for the remainder of the experiment; this regime is designed to simulate post-extrusion conditions, where the ink resists gravitational stresses and retain its structure. Upon cessation of shear, the precursor’s apparent viscosity increases until it reaches an equilibrated stagnant value within 40-60 seconds due to the thixotropic behavior of the precursor as the elastic particle gel network reforms, agreeing with the timescale found through creep and recovery. This recovery timescale is more clear in Figure S6d, which recasts the data in Figure S6c to focus on the third shear-rate step. However, viscosity does not completely recover to its pre-extrusion value; the precursor’s particle gel is disrupted during extrusion but is unable to recover to its original state post-extrusion. While the relationship between rheological changes and microstructural changes is not fully understood, the precursor ink’s reduction in viscosity in high-shear environments is tied to changes in the precursor's fumed silica gel network, which gradually recovers when the high shear is removed.^[2]^

**Surface microstructure of 3D printed bicontinuous emulsion gels**

We print a bicontinuous emulsion gel with a vertical rectangular prism geometry. After the prism is printed, the oil phase is polymerized via UV curing, then washed in ethanol and dried to remove any excess water and solvent from the material. One of the four walls is cleaved from the structure and its surface is characterized via SEM in Figure S7. When untreated, minimal microstructures are observed; this occurs due to drying out of the emulsion before the UV curing process, resulting in the formation of nanoparticle-laden oil domains on the surface, as seen in Figure S7a. However, by removing the particles via silica etching, the bicontinuous porous structure is revealed, indicated in Figure S7b. The structures are similar to those of the cross-sectional images in Figure 3C, suggesting structural uniformity throughout the 3D printed monolith.


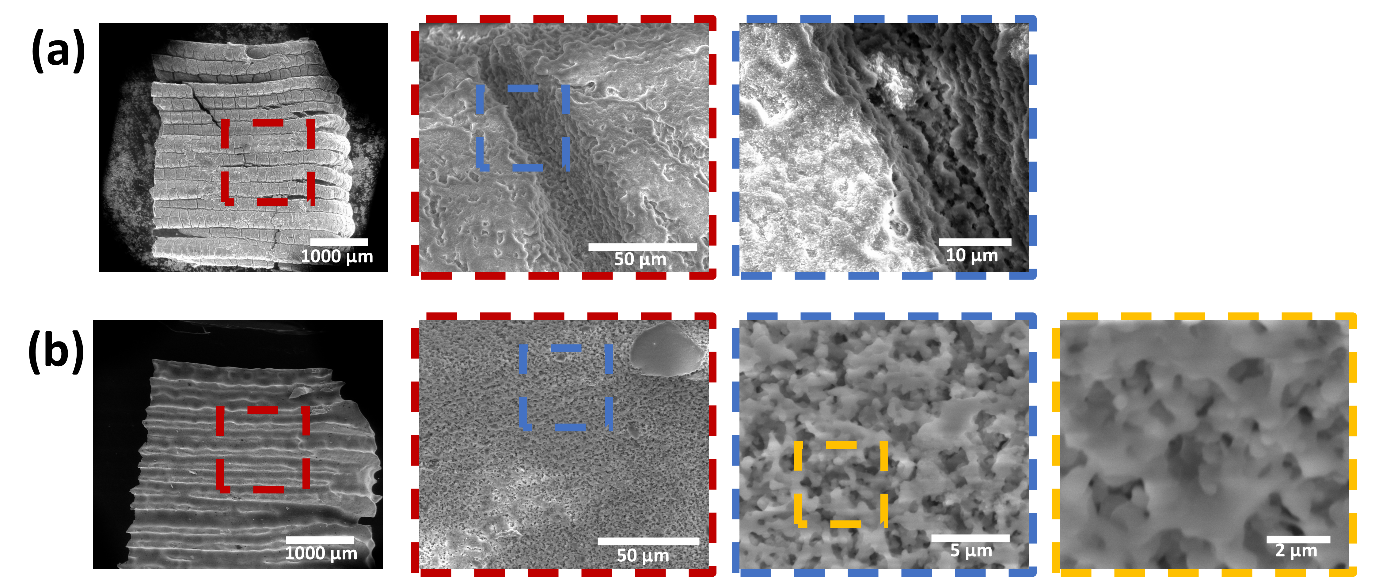


**Figure S7.** Surface microstructure of 3D printed bicontinuous emulsion gels**.** a) Surface SEM series of a 3D printed bicontinuous emulsion gel. The surface is covered with a polymer-particle crust. b) Surface SEM series of a 3D printed bicontinuous emulsion gel after removal of fumed silica particles, revealing the bicontinuous polymer-pore microstructure.

**3D Reconstruction of bicontinuous morphology**

To further demonstrate the bicontinuous morphology, we observe the three-dimensional structure of a printed structure. We print a bicontinuous gel with a rectangular prism shape. The printed structure is UV cured two minutes after 3D printing; the oil phase is crosslinked into a polymeric phase containing fluorescent Nile red. The structure is then washed in ethanol and dried, to remove excess water and solvent. A wall from the 3D printed structure is cleaved, then fumed silica particles are dissolved by submerging the cleaved structure in 1m NaOH overnight.

We image the cleaved portion of the 3D printed bicontinuous gel via CLSM. While imaging, the structure is immersed in DEP. The refractive index of DEP is similar to that of the polymer phase; thus the structure becomes transparent, facilitating confocal microscopy. The structure is oriented such that the face of the cleaved wall faces the microscope lens. We image a z-stack of the structure, consisting of 25 image slices each separated by 0.5 µm. The z-stack is rendered into a 3D model, as shown in Figure S8. 3D reconstruction is performed through Fiji ImageJ. The z-stack is binarized (via Process 🡪 Binary 🡪 Make Binary). The binarized z-stack is then imaged through the 3D Viewer Plugin (via Plugins 🡪 3D Viewer).


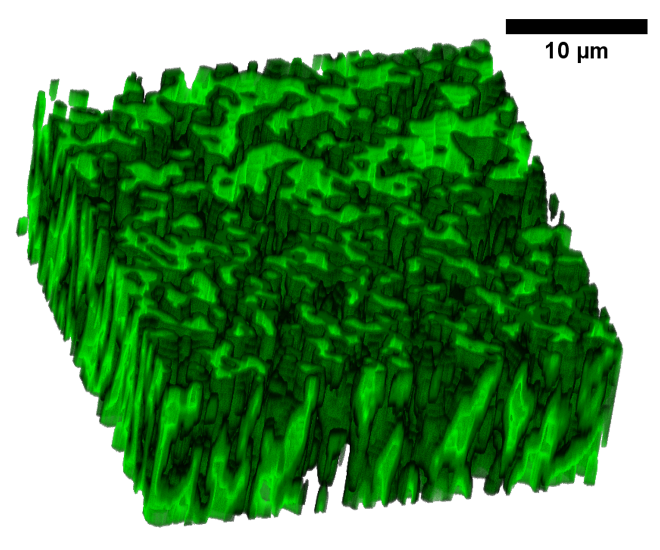


**Figure S8.** 3D reconstruction of bicontinuous gel morphology. The green signal corresponds to polymeric domains containing Nile red, and the empty regions correspond to the domain that was filled with water.

**Pore phase continuity of a polymerized 3D printed bicontinuous emulsion**

We test the pore continuity of a polymerized 3D printed bicontinuous emulsion by visually monitoring capillary-driven flow of DEP within the pores. Since DEP has a similar index of refraction of poly(HDA), the polymerized construct transitions from a visually white to a transparent state as DEP permeates across its pores (there is a slight pink hue due to the presence of Nile red). To do this, we create a bicontinuous emulsion gel via DIW, with a macroscale geometry of a wall. The emulsion gel is polymerized via UV curing, then dried, producing a porous wall consisting of polymer and pore phases. One end of the wall is placed on a coverslip, while the other end rests over a pipette tip. DEP is added dropwise to the coverslip end; the DEP permeates into the wall’s pores via capillarity, and the DEP moving front is observed visually across one hour, as seen in Figure S9. DEP is able to rise across the 3D printed wall, indicating continuity of the pore phase across printed layers.


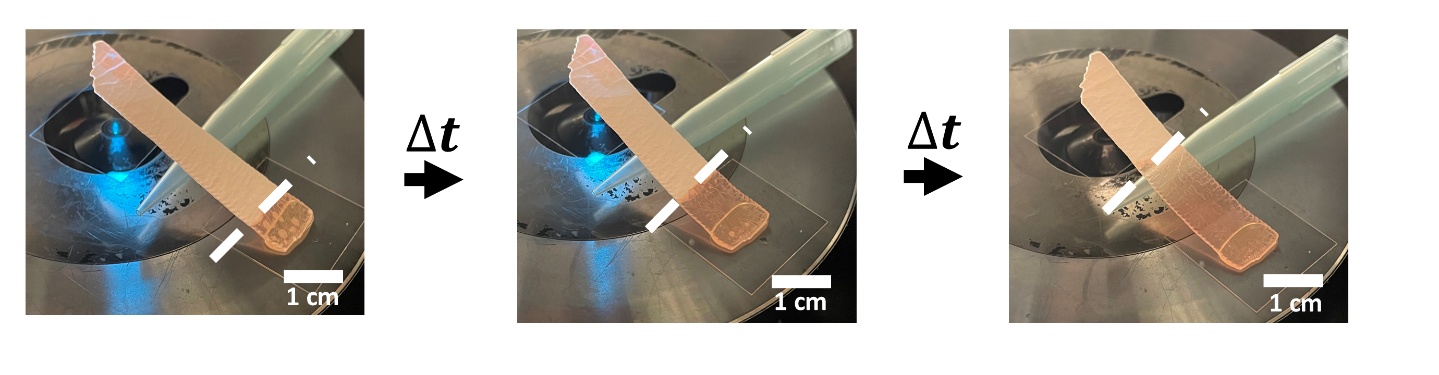


**Figure S9.** Pore phase continuity of a polymerized 3D printed bicontinuous emulsion**.** Permeation of DEP across a polymerized emulsion gel is monitored for an hour ($\Delta t=30$ min); the porous 3D printed wall is visually monitored as it transitions from an opaque state to a transparent state. Rising of the DEP front indicates continuity of the pores in the 3D printed wall.

This structure, and others reported in this manuscript, are fabricated continuously across printed filaments layer by layer, then polymerized through a single UV curing step after the entire structure is printed and quenched. This process produces materials with bicontinuous morphology that extends across printed layers. Such continuity in microstructure is lost, however, when structures are fabricated in a “print-cure-print” approach, where layers are cured before the next set of layers are printed. This is observed by printing a rectangular prism, similar to that presented in Figure 3. However, in this case, the first 10 layers are printed and subsequently UV cured; then 10 additional layers are printed on top and subsequently UV cured. We find that the upper portion of this “print-cure-print” generated structure does not adhere to the lower portion; when picking up the printed structure with tweezers, the top 10 layers cleanly detach from the bottom 10 layers along their printed interface (see Video S2). These findings indicate that the “print-cure-print” interferes with formation of interlayer bicontinuous morphology; this suggests that the ethanol in freshly extruded filaments plays an important role in connecting the microstructured bicontinuous domains of adjacent printed layers.

**Interfacial tension at the HDA/water interface**

The bicontinuous emulsion gel precursor ink is a one-phase miscible ternary liquid mixture, with a high concentration of ethanol, which acts as a co-solvent. When the ink is extruded, ethanol evaporates as it is exposed to the open air. As the co-solvent concentration decreases due to evaporation, the extruded ink undergoes phase separation into an oil-rich domain and a water-rich domain, whose concentrations are determined by their phase diagram tie-lines. Phase separation introduces an HDA/water interface within the system, and the interfacial tension between the two phases is influenced by the compositions of the two domains.

We use pendant drop analysis to explore how interfacial tension changes as a function of ethanol concentration. To do so, we form HDA pendant droplets suspended in binary blends of water and ethanol, where HDA is the heavier component and the water/ethanol blend is the lighter component. A camera captures the shape of the HDA droplet across a 300-second interval, which is used to calculate the Bond number and interfacial tension. The measurements are repeated as the concentration of ethanol in water is increased from 0% to 30%. We report the average interfacial tension value across the 300-second interval at each concentration. We perform these sets of experiments using pure HDA and hydrated HDA, as shown in Figure S10. Hydrated HDA is prepared by vortexing pure HDA with the water/ethanol mixture for 60 seconds, before centrifuging the mixture and extracting the HDA-rich phase before pendant drop analysis.


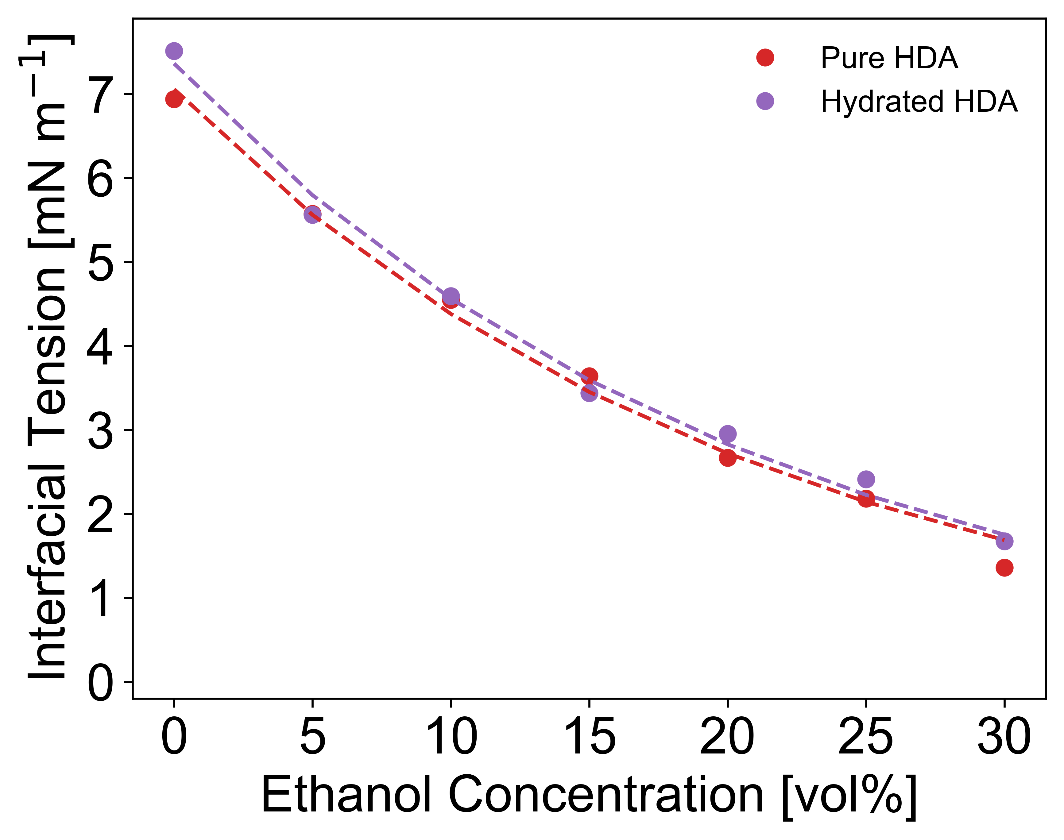


**Figure S10.** Interfacial tension at the HDA/water interface. The interfacial tension between pure HDA (red) and hydrated HDA (purple) droplets suspended in water/ethanol mixtures is measured via pendant drop analysis. As the solvent concentration decreases, the interfacial tension between oil and water phases increases. The dashed line represents a fit to an exponential decay.

We find that the interfacial tension decreases as the concentration of ethanol increases. This behavior is similar to other reported bicontinuous emulsion systems.^[3]^ As co-solvent evaporates from the mixture and ethanol concentration decreases, the oil-rich domain and water-rich domain become increasingly dissimilar as the system quenches deeper beneath the binodal curve in the phase diagram. Therefore, in VIPS-generated bicontinuous emulsion gels, the interfacial tension increases during ethanol evaporation, promoting interfacial particle attachment and jamming.

We note that these experiments are performed under static conditions, where solvent concentration is held constant during the measurement. However, the interfacial dynamics in bijel formation are much more complex. The precursor begins as a one-phase miscible blend and phase separates into two phases: an oil-rich domain and a water-rich domain, which quenches deeper across time. Since the co-solvent concentration decreases with time as ethanol evaporates, the interfacial tension increases across the quenching period.

**DIW extrusion printing map**

For successful DIW, printing parameters must be optimized to ensure proper extrusion. The printing parameters we focus on are print speed, printing pressure, and nozzle diameter. The print speed is the translational velocity of the printhead as it moves around the print bed; the printing pressure is the pressure applied to the ink loaded into the syringe to induce flow through the nozzle, and the nozzle diameter measured as the inside diameter at the point of exit.

We print a 40 mm line with a silica concentration of 12.5 wt% in the precursor ink at different combinations of print speed and printing pressure, with nozzle diameters of 250 µm and 400 µm, as shown in Figure S11a and S11b, respectively. Print speeds are tested at 10, 20, 30, and 40 mm s^-1^. Printing pressures are tested between 5 and 90 psi, in increments of 5 psi. Each line is then classified into one of four extrusion regimes: no extrusion, under-extrusion, proper-extrusion, and over-extrusion. We include an example of each of these extrusion behaviors in Figure S11c; an image of each line is captured using a USB digital microscope mounted to the print head. If the ink fails to exit the nozzle, it is classified as “no extrusion”. If the ink drips out from the nozzle, it is classified as “under-extrusion”. If the ink prints into a straight filament, it is classified as “proper-extrusion”. And if the filament buckles, curls, or globs, then it is classified as “over-extrusion”.


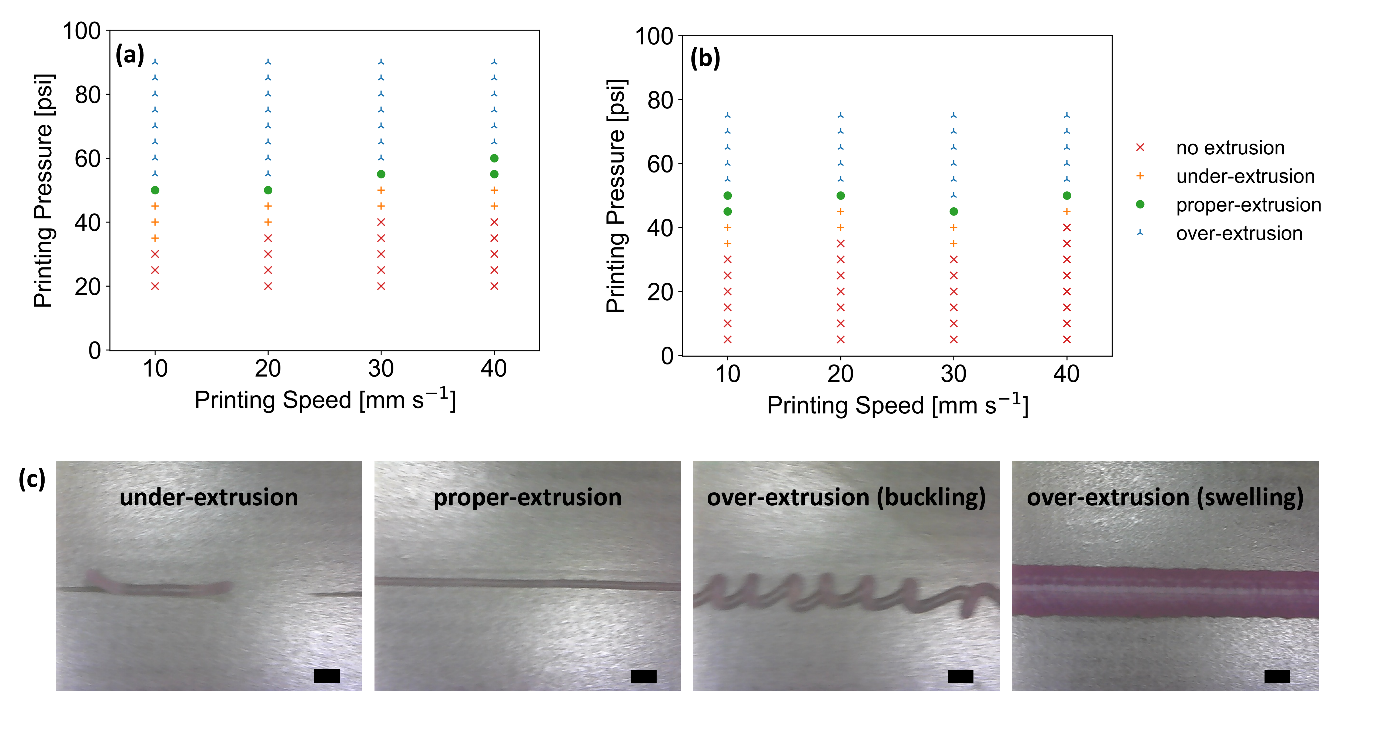


**Figure S11.** Printing parameter maps of the printable bicontinuous emulsion precursor ink. a) Extrusion regime diagram with a nozzle diameter of 250 µm. b) Extrusion regime diagram with a nozzle diameter of 400 µm. c) Exemplary images of filaments for each extrusion behavior classification (scale bar = 1 mm).

The print map reveals there is a minimum pressure of around 30 psi to induce flow out of the nozzle. This minimum pressure also depends on the print speed, as high print speeds result in shorter print durations, thus there is less time for the fluid to extrude from the nozzle, requiring a higher pressure needed for extrusion. As the pressure increases, the ink begins to flow. To form a proper filament, the speed of ink extrusion must roughly match the print speed. If the ink extrusion is too slow relative to the print speed, the filament may only drip out of the nozzle or the printhead may pull the filament apart into smaller fragments. If the ink extrusion is too fast relative to the print speed, the filament may buckle, curl, spiral, or swell as it extrudes. We find that there are only narrow combinations of print speed and pressure that produce properly extruded filaments. As the print speed increases, a higher printing pressure is needed to ensure proper extrusion. Further, we find that using larger nozzles results in a reduced printing pressure for proper extrusion, which is more pronounced at higher print speeds. This occurs due to a reduction in the nozzle’s frictional losses the ink must overcome in order to extrude, which has a direct relationship with print speed and has an inverse relationship with nozzle diameter.

**Mechanical properties of cured prints**

An Instron 685C-5 universal testing system is used for compression testing, equipped with 50 mm diameter compression plates. A filled rectangular prism is printed via DIW using the precursor ink. The print is quenched via ethanol evaporation, then UV cured and dried, to produce a porous polymeric material. A caliper is used to record the dimensions of the cured structures. The material is placed on the compression plate, and the applied load is measured as the function of the displacement between the two plates. Load is then expressed in terms of stress, using the relation $\sigma=F/{(W*L)}$, where $\sigma$ is the stress (Pa), $F$ is the load (N), while $W$ and $L$ are the prism’s width and length (m), respectively. Strain is calculated using the displacement, with the relation $\varepsilon=100*x/H$, where $\varepsilon$ is the strain (%), $x$ is the displacement (m), and $H$ is the prism’s height (m). Using these relations and measurements, we explore the mechanical properties of the material in Figure S12.


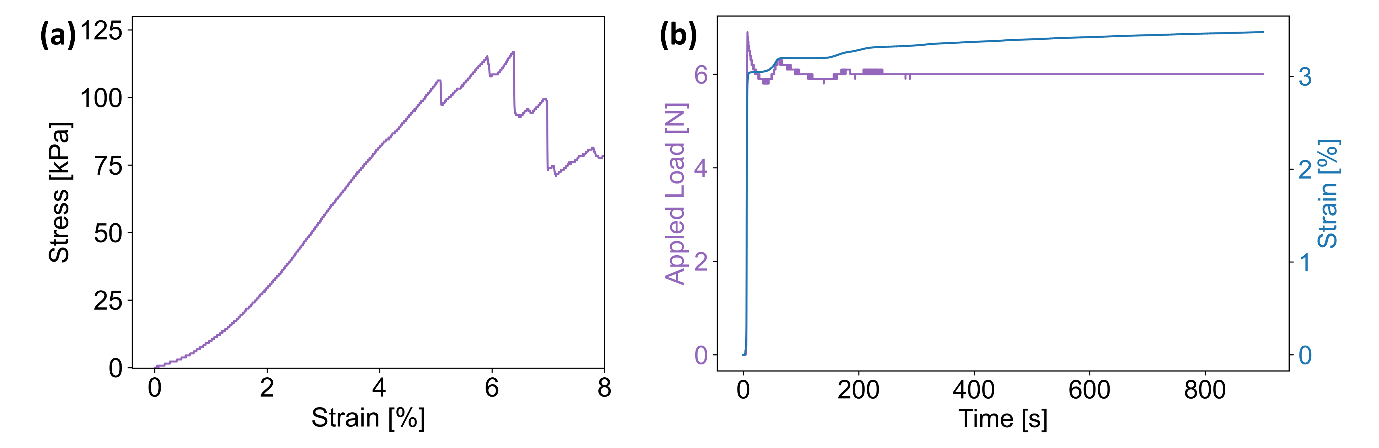


**Figure S12.** Mechanical properties of cured prints**.** a) The stress-strain curve is measured via compression testing of a cured print with a filled rectangular prism geometry. b) Creep tests measure the material’s strain as a function of time while under a constant load of 6 N.

The stress-strain relationship of the material exhibits that of a typical brittle material. The material’s stress increases with the applied strain, until a maximum stress is reached at a strain of around 6%. Due to the brittle nature of the material, there is a lack of plastic deformation, and thus, it fractures abruptly at this point. As a result, we measure the yield strength as the maximum compressive strength which is found to be around 116 kPa. The compressive modulus is calculated as the linear slope at low strains (averaged between strains of 2 and 4%), with a value of around 2.5 MPa.

We also employ creep testing under constant load to explore failure dynamics, as shown in Figure S12. A load is applied at a rate of 1 N s^-1^, until a setpoint of 6 N is reached after 6 seconds, which is held for 15 min. The load and displacement are measured as a function of time. Initially, the strain increases as the displacement increases until the 6 N load is applied. The strain reaches two plateaus within the first three minutes, as the applied load equilibrates to the 6 N set point. Beyond 3 min, the material experiences secondary creep, with a minimum creep rate of 0.81 % hr^-1^. The material does not fracture across the 15-minute test; thus, tertiary creep is not detected.

**Mass loss in 3D printed bicontinuous emulsion gels**

Once a filament is extruded, its mass begins to decrease, as ethanol evaporates from the precursor ink spontaneously in ambient conditions. To quantify this, we use a balance to monitor the mass of a printed filament extruded into a planar sheet geometry for three minutes after printing. The mass of the sheet decreases by 5%, due to the evaporation of ethanol, as seen in Figure S13. This is sufficient to trigger phase separation within the film. Further, evaporation is influenced by a variety of factors (such as temperature, humidity, airflow, surface area, etc.).


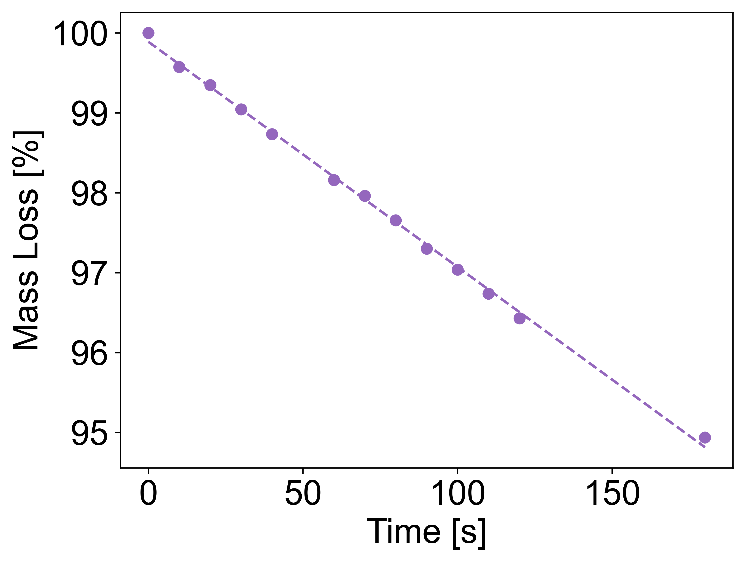


**Figure S13.** Mass loss of 3D printed bicontinuous emulsion gels. The mass of a 3D printed planar sheet is measured after the printing process is completed. The printed material’s mass decreases at a rate of 4.6 mg min^-1^ due to ethanol evaporation, which drives phase separation to generate the bicontinuous emulsion structure during the quenching process.

We also use a caliper to measure the dimensions of a printed bicontinuous emulsion across the quenching and curing processes. In this example, we print a hollow rectangular prism; once printing is complete, the dimensions are measured as 21.08 x 21.48 x 10.11 mm (W x L x H). The prism is then quenched for two minutes as ethanol evaporates. The dimensions are measured again as 20.80 x 20.60 x 9.86 mm, indicating a slight reduction upon quenching. The prism is then placed under UV irradiation to cure the oil phase. After a one-minute quenching period, the dimensions are measured as: 19.22 x 19.80 x 8.04 mm.

**Video S1.** DIW failure using conventional VIPS emulsion precursor

**Video S2.** Interlayer adhesion of “print-cure-print” fabricated structures

**Video S3.** 3D printing of a rectangular prism

**Video S4.** 3D printing of a starfish

**References**

1. G. Di Vitantonio, D. Lee and K. J. Stebe, “Fabrication of Solvent Transfer-Induced Phase Separation Bijels with Mixtures of Hydrophilic and Hydrophobic Nanoparticles,” *Soft Matter* 16, no. 25, (2020): 5848, https://doi.org/10.1039/D0SM00071J

2. R. G. Larson and Y. Wei, “A Review of Thixotropy and Its Rheological Modeling,” *Journal of Rheology* 63, no. 3, (2019): 477, https://doi.org/10.1122/1.5055031

3. H. Siegel, M. De Ruiter, T. H. R. Niepa and M. F. Haase, “The Effect of Charge Screening for Cationic Surfactants on the Rigidity of Interfacial Nanoparticle Assemblies,” *Journal of Colloid and Interface Science* 678, (2025): 201, https://doi.org/10.1016/j.jcis.2024.08.133
